# Mapping the computational similarity of individual neurons within large-scale ensemble recordings using the SIMNETS analysis framework

**DOI:** 10.3389/fnins.2025.1634652

**Published:** 2025-08-14

**Authors:** Carlos E. Vargas-Irwin, Jacqueline B. Hynes, David M. Brandman, Jonas B. Zimmermann, John P. Donoghue

**Affiliations:** ^1^Department of Neuroscience, Brown University, Providence, RI, United States; ^2^Robert J. and Nancy D. Carney Institute for Brain Science, Brown University, Providence, RI, United States; ^3^VA Center for Neurorestoration and Neurotechnology, VA Providence Healthcare System, Providence, RI, United States; ^4^Department of Neurological Surgery, UC Davis, Sacramento, CA, United States; ^5^Wyss Center, Geneva, Switzerland; ^6^Department of Engineering, Brown University, Providence, RI, United States; ^7^Department of Cognitive and Psychological Sciences, Brown University, Providence, RI, United States

**Keywords:** neural ensembles, dimensionality reduction, clustering, neuronal networks, subnets

## Abstract

The expansion of large-scale neural recording capabilities has provided new opportunities to examine multi-scale cortical network activity at single neuron resolution. At the same time, the growing scale and complexity of these datasets introduce new conceptual and technical challenges beyond what can be addressed using traditional analysis techniques. Here, we present the Similarity Networks (SIMNETS) analysis framework: an efficient and scalable pipeline designed to embed simultaneously recorded neurons into low dimensional maps according to the intrinsic relationship between their spike trains, making it possible to identify and visualize groups of neurons performing similar computations. The critical innovation is the use of pairwise spike train similarity (SSIM) matrices to capture the intrinsic relationship between the spike trains emitted by a neuron at different points in time (i.e., different experimental conditions), reflecting how the neuron responds to time-varying internal and external drives and making it possible to easily compare the information processing properties across neuronal populations. We use three publicly available neural population test datasets from the visual, motor, and hippocampal CA1 brain regions to validate the SIMNETS framework and demonstrate how it can be used to identify putative subnetworks (i.e., clusters of neurons with similar computational properties). Our analysis pipeline includes a novel statistical test designed to evaluate the likelihood of detecting spurious neuron clusters to validate network structure results. The SIMNETS framework provides a way to rapidly examine the computational structure of neuronal networks at multiple scales based on the intrinsic structure of single unit spike trains.

## 1 Introduction

The neural processes underpinning complex sensory, cognitive, and behavioral phenomena engage complex activation patterns across brain-wide networks (Fox et al., 2005; Greicius et al., 2003; Raichle et al., 2001; Sporns, 2013; Thomas Yeo et al., 2011). Within large-scale networks, smaller clusters of computationally interrelated neurons have been proposed to embody fundamental input–output processing modules (‘subnetworks’) responsible for perceptual integration, dexterous motor control, and memory storage or retrieval (Briggman et al., 2006; Harris, 2005; Truccolo et al., 2010; Yuste, 2015; Zagha et al., 2015). While recent technological advances have made it possible to record from ever larger neuronal populations at single neuron resolution (Buzsáki, 2004; Chung et al., 2022; Donoghue, 2002; Maynard et al., 1997; Paulk et al., 2022; Steinmetz et al., 2021; Steinmetz et al., 2018), progress in understanding the computational organization of large networks has been hindered because of the lack of appropriate conceptual and technical frameworks that are mathematically principled; amenable to a range of statistical tools, flexible, scalable; and fast enough to work on large datasets (Brown et al., 2004; Gao and Ganguli, 2015; Paninski and Cunningham, 2018; Stevenson and Kording, 2011; Zador et al., 2022). Objectively identifying and characterizing subnetwork structure within and across brain areas would greatly simplify the process of tracking information flow within cortical circuits, modeling multi-scale neural dynamics, and understanding the general principles of neural information processing (Alivisatos et al., 2013; Kohn et al., 2020; Urai et al., 2022; Yuste, 2015).

How can computational subnetworks be identified? In cortical areas where the tuning properties of individual neurons are well-established, such as the primary visual (V1) and motor (M1) regions, the most straightforward approach to assessing computational inter-relationships would be to calculate tuning parameter similarities across neurons (Berman et al., 1987; Georgopoulos et al., 1982; Livingstone et al., 1996). However, simple parametric tuning models often fail to capture the temporal complexities of single neuron outputs and can perform poorly or even break down entirely under more ecologically or ethologically relevant experimental conditions (David et al., 2004; Hosman et al., 2021; Olshausen and Field, 2005; Pospisil and Bair, 2021). Moreover, classic single-neuron tuning function estimation techniques can miss or “average away” computationally-relevant features of individual spike trains (Churchland and Shenoy, 2007; Cunningham and Yu, 2014; Malik et al., 2011).

A variety of useful and important tools have been developed to study coordinated neuronal activity based on the underlying premise that similar spike patterns shared by a pair of neurons imply similar information processing properties (Abeles and Gat, 2001; Aertsen and Gerstein, 1985; Amarasingham et al., 2012; Gerstein et al., 1985; Gerstein and Michalski, 1981; Grün et al., 2002a; Kiani et al., 2015; Lopes-dos-Santos et al., 2013). These methods group neurons according to the similarity of spiking statistics (e.g., coincident spiking or correlated spike rate fluctuation). However, the mechanistic origin and computational significance of these correlations have proven more complex than initially theorized and are not fully understood (Brody, 1999; Cole et al., 2016; de la Rocha et al., 2007; Friston, 2011; Roudi et al., 2015; Smith and Kohn, 2008; Soudry et al., 2013). This approach has limited the utility and explanatory power of inter-neuronal correlations as a means for establishing the computational relatedness of neurons within a common feature space. These interpretational issues are further confounded by the significant statistical and computational challenges that come with implementing these methods at scale (Amarasingham et al., 2012; Brody, 1999; Cohen and Kohn, 2011; de la Rocha et al., 2007; Soudry et al., 2013).

Here, we present SIMNETS, an unsupervised relational analysis framework designed to generate low-dimensional neuron maps that quantify and support a multi-scale view of the computational similarity relations among individual neurons. For the purposes of our analysis, we define computation as the mapping of a set of inputs to a set of outputs according to a given set of rules (Agüera y Arcas et al., 2003; Kanerva, 2009). Under these conditions, the computational equivalence of any two neural systems should not be sought solely in the actual pattern of their spiking outputs, but *in the relations of their outputs to one another within each system* (Kanerva, 2009; Shepard and Chipman, 1970). Based on this principle, SIMNETS compares neurons using the intrinsic relational structure of their firing patterns, represented by a spike train similarity (SSIM) matrix that captures the relative changes in activity across a set of predetermined time windows. In this way, each SSIM matrix can be considered to represent the “output space” of a neuron, which serves as a computational “fingerprint” within the context of a given dataset (Vargas-Irwin et al., 2015a). By employing the single neuron SSIM matrix representation of individual neuron output spaces, we can efficiently quantify the computational relationships among all neuron pairs within a population of concurrently recorded single units using relatively straightforward techniques, such as vector correlation or regression. As we will demonstrate, this type of inter-neuronal 2nd-order spike train comparison—the Computational Similarity (CS) score—becomes an effective means for identifying neurons that share computationally similar output spaces, even in cases where diverse encoding strategies are employed. For instance, consider two neurons responding to a specific subset of trials – one through elevated firing rates and the other through specific spike timing sequences. Despite distinct coding mechanisms, an appropriate similarity metric can capture the underlying congruency in trial-dependent response modulation. This approach enables the assessment of more nuanced computational relationships among biophysically diverse neurons. In short, neurons that have the same pattern of change in their responses across trials (i.e., intrinsics spike train similarity structure) are considered to be performing similar computations. For very large neuron populations, we can simplify the population-level interpretation of the set of CS scores by projecting the high-dimensional CS matrix into a lower-dimensional coordinate space, such that each point in this latent space represents a neuron and the distance between them corresponds to their degree of computational similarity. The geometric structure of the low-dimensional neuron embedding can greatly facilitate the identification of discrete clusters and gradients of functionally related neurons. As we will demonstrate, this general framework can efficiently scale to large numbers of trials and neurons, making the method particularly suited for examining large-scale neural recording datasets that were collected under ecologically or ethologically relevant experimental conditions.

## 2 Methods

**Figure 1:**
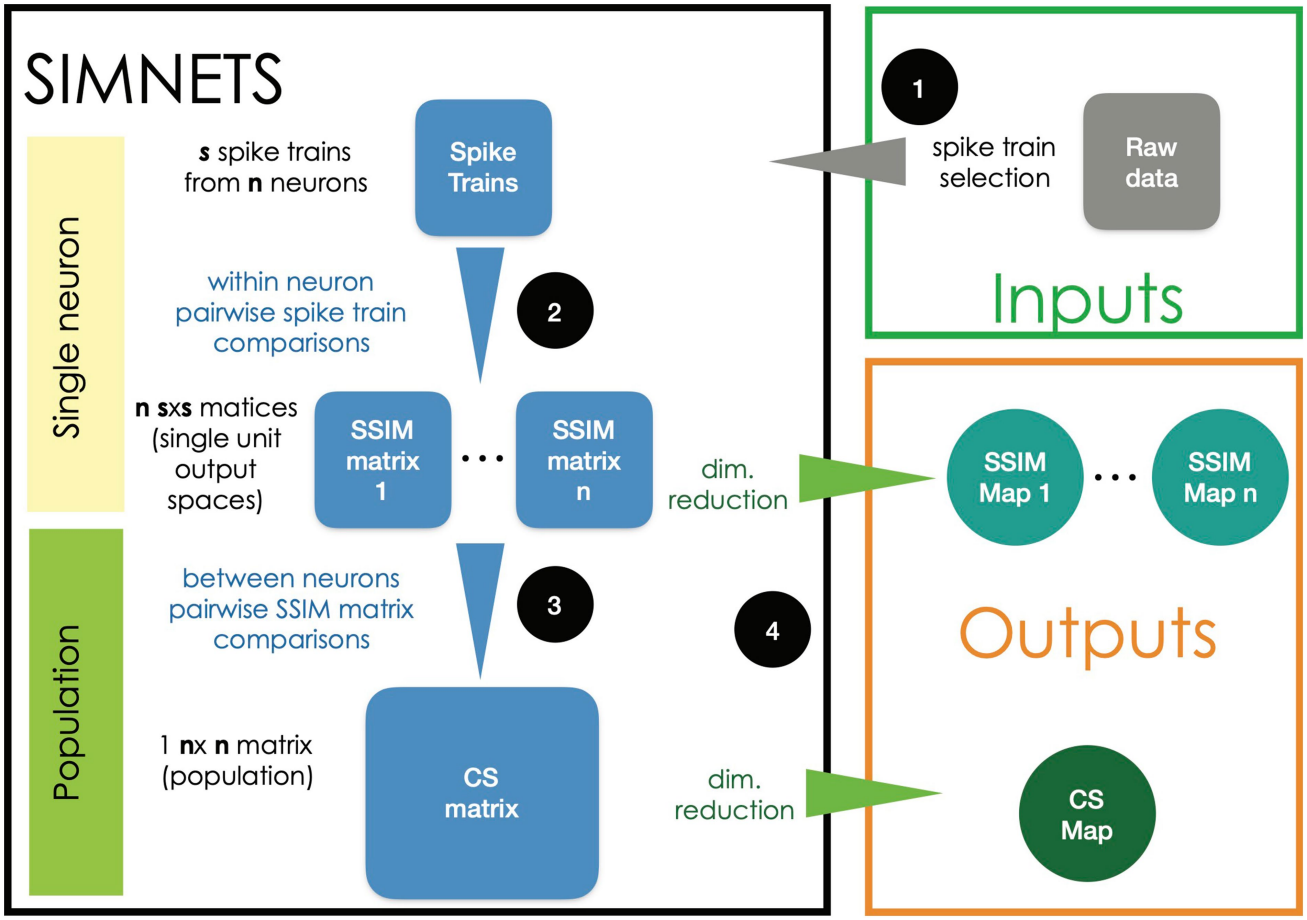
Flow diagram of the SIMNETS analysis framework. See Methods section for details.

**Figure 2:**
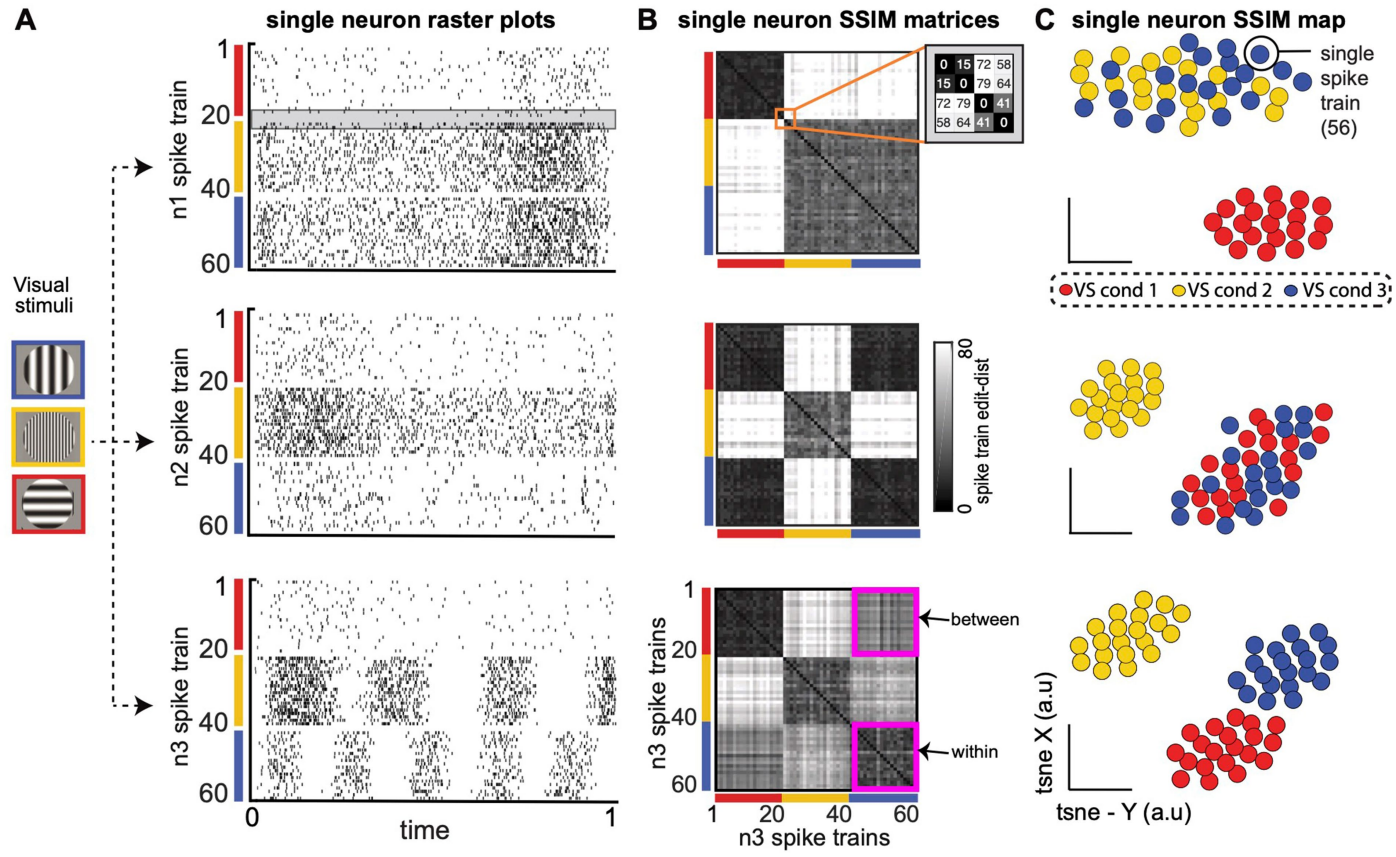
Single neuron Spike train Similarity (SSIM) matrices capture the intrinsic structure of each neuron’s spike train output space (OS) without the need for extrinsic labels or tuning models. **(A)** Spike train raster plots for three computationally distinct LNP model V1 neurons (*n1, n2, n3*) during simulated receptive field stimulation (Meyer et al., 2016). Three visual stimulus (VS) conditions include a vertically oriented grating of low spatial frequency (SF; red, cond 1); a horizontally oriented, high SF grating (yellow, cond 2); and a horizontally orientated, low SF grating (blue, cond 3). Gray shaded region in the n1 raster plot highlights a similar pair of spike trains from VS condition 1 (S19–S20) and a similar pair from VS condition 2 (S21–S22) that will be highlighted in **B**. **(B)** Neuron n1 (top), n2 (middle), n3 (bottom) *single neuron SSIM matrices* depict the SxS pairwise similarities among a neuron’s spike trains (a within-neuron comparison). Similarities are described in terms of “cost-based” edit distance (grayscale color bar), where smaller distance values correspond to similar spike trains (black, lowest edit-cost), and increasingly large distance values are increasingly dissimilar spike train outputs (white, larger edit-cost). The orange square in the top row (n1 SSIM matrix) highlights the set of distance values for the example spike trains S19–S22 [highlighted in **(A)**]. The magenta squares in the (bottom row; n3 single neuron SSIM matrix) highlight the matrix region that contains containing “within-condition” edit-distances (n3 cond-1, n3 cond-1) and “between-condition” distances (n3 cond-1, n3 cond-3). **(C)** Three single neuron SSIM maps, the low-dimensional projection of each neuron’s single neuron SSIM matrix [from **(B)**]. The intrinsic geometry of each neuron’s spike train output space provides a richer and intuitive representation of trial-by-trial single-neuron functional responses than standard parametric or statistical descriptions of single neuron responses (e.g., trial-averaged tuning curves).

The flow chart presented in Figure 1 provides a general overview of the main steps used for analyzing neural ensemble data using the SIMNETS framework:

1. Selecting spike trains. Time series data representing the activity of N simultaneously recorded neurons (e.g., N spike time sequences) are split into S equal-duration time segments corresponding to experimentally relevant periods of interest.
2. Generating a Spike train Similarity (SSIM) matrix for each neuron. Use an appropriate metric (e.g., VP edit-distance; Victor and Purpura, 1996) to calculate the intrinsic pairwise similarities among each neuron’s set of S spike trains, resulting in a set of N SxS SSIM matrices.
3. Calculating the NxN Computational Similarity (CS) matrix. Calculate the pairwise similarities (e.g., Pearson’s correlation *r*) among all pairs of single neuron SSIM matrices. The resulting set of CS scores is represented as a NxN population CS matrix, where each entry depicts the similarities among the output space of a neuron pair and depicts the overall pattern of relationships among the neurons for the experiment.
4. Visualizing results using dimensionality reduction (DR). An appropriate DR method (t-distributed stochastic neighbor embedding [t-SNE], multidimensional scaling [MDS], etc.) can be applied to the SSIM matrices generated in step 2 to visualize the single neuron SSIM matrix as SSIM maps (example provided in Figure 2C) where each point represents an individual spike train, and the distance between them represents their relative similarity (Maaten and Hinton, 2008). This step makes it easier to interpret the neuron’s relationship to task variables (e.g., stimulus inputs, movement types, outlier trials) and ultimately their computational role within the context of higher-level network structures. Applying DR to the CS matrix generated in step 3 will produce a low-dimensional CS map, with each point corresponding to an individual neuron, and the distance between them corresponds to their relative computational similarity (i.e., the similarity between their respective SSIM matrices).

**Figure 3:**
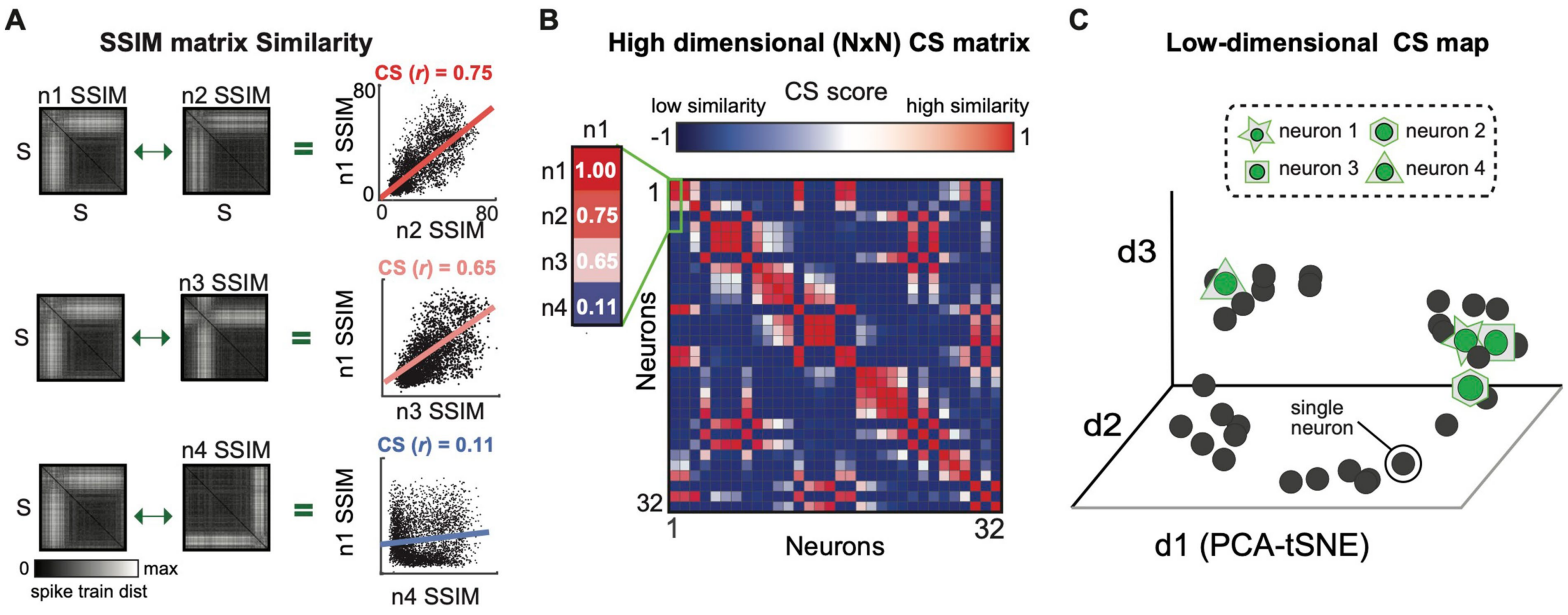
The similarity between the output spaces (SSIM matrices) of all individual neurons is represented as a computational similarity (CS) matrix and low-dimensional CS map. **(A)** The Computational Similarity matrix is calculated by correlating each pair of SSIM matrices, illustrated for three example neuron pairs. The regression line depicts the relationship among the VP distance values for each pair of neurons. The similarity of the top two matrices (n1 v n2) and the differences in the bottom two (n1 v n4) is captured in their respective Pearson correlation values. **(B)** Population CS matrix, highlighting the correlation values shown in **A**. **(C)** CS Map of neuron similarity across the population obtained by applying DR to the CS matrix. Each cluster in the CS map reflects a group of neurons with similar computational properties (i.e., a potential sub-network).

**Figure 4:**
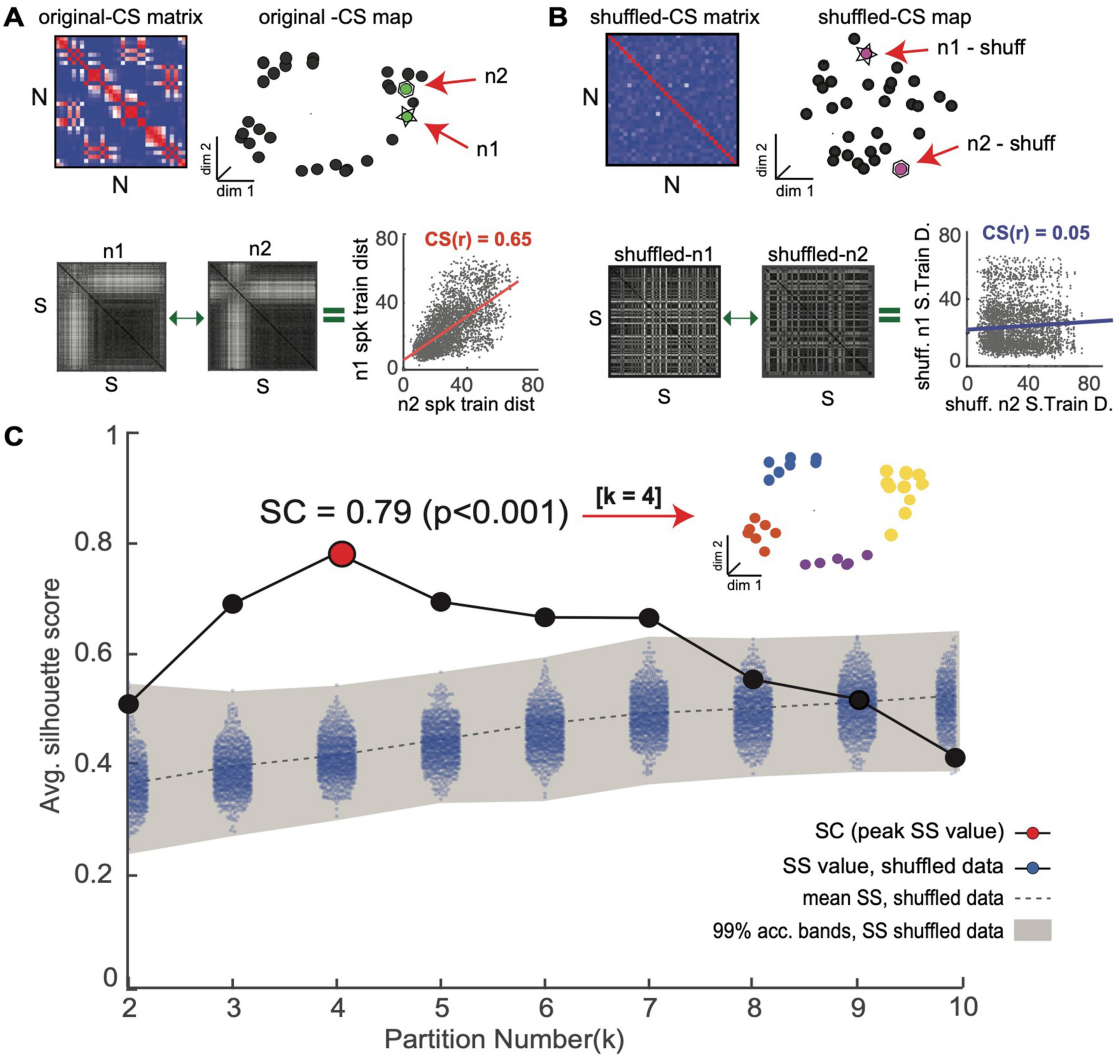
Novel shuffle-based silhouette analysis is used to detect and validate CS neuron clusters within an observed CS neuron map. The goal is to determine if an observed CS neuron map contains statistically meaningful neuron clusters. **(A)** Top: population CS matrix and map for an example simulated neuron population (same as Figure 3). Bottom: Two example single neuron SSIM matrices (n1 and n2) for a neuron pair with a high CS score (CS(r) = 0.65). **(B)** Top: Surrogate CS matrix and map generated using CS neuron scores obtained after independently randomizing spike train order in all single neuron SSIM matrices. Bottom: SSIM matrices for the neurons shown in **A** after shuffling display reduced CS scores. **(C)** The shuffling procedure is repeated many times (e.g.,10,000) to generate an empirical chance distribution of silhouette values and obtain acceptance bands. Shading denotes the 99% confidence interval of surrogate data, which serves as an acceptance band for the null hypothesis (see Amarasingham et al., 2012; Schemper, 1984 for further discussion). The silhouette values of the original data (without shuffling) are overlaid in black. The highest silhouette value (highlighted in red) is used to select the optimal number of clusters. Clusters corresponding to the highest (Ksc = 4) are shown in the inset.

The CS map is the main output of the algorithm, capturing the computational organization of the ensemble in a latent space representation and serving as a starting point for further analysis aimed at identifying groups of neurons with similar computational properties. The following sections and figures describe specific steps (Figures 2, 3) and subsequent analysis (Figure 4) in more detail.

### 2.1 Representing single neuron output spaces using SSIM matrices

One of the key innovations of the SIMNETS framework consists of using single neuron SSIM matrices as a “computational fingerprint” to represent the intrinsic structure of the output space of individual neurons. This is accomplished by performing within-neuron measures of pairwise spike train similarity across all spike trains for each neuron, resulting in an SxS single neuron SSIM matrix for each of the N neurons (Figure 1 step 2; Figure 2B). Multiple approaches to estimate the similarity between pairs of spike trains have been proposed (Paiva et al., 2010) and could be used for this step. Here, we use the spike train metric proposed by Victor and Purpura (Houghton and Victor, 2010; Victor and Purpura, 1997; Victor and Purpura, 1996; See Box 1 for description).

Box 1 **Victor and Purpura edit-based spike train metric**

This VP metric is one type of edit-distance measure to quantify the differences between pairs of spike trains. The method computes the total “cost” of transforming one spike train into another through a series of elementary operations (Victor, 2005; Victor and Purpura, 1996). These elementary operations include (1) inserting a spike, (2) deleting a spike, and (3) shifting a spike in time. Inserting or deleting a spike has a cost of c = 1 and shifting a single spike in time has a cost proportional to the amount of time that it is moved (c = qΔ t). The set of edits-steps associated with the minimum total edit-cost defines the shortest path or ‘distance’ (D) between two points (spike trains) in the neuron’s spike train metric-space. The q parameter, a measure of temporal precision of the comparison, influences the relative importance of spike count and spike time differences when assessing spike train similarities. When q = 0, the cost of shifting a spike to the desired location will always be cheaper than deleting and re-inserting a spike in a spike train. Thus, for D(q = 0), the minimum cost is simply the difference in the number of spikes between the spike trains (essentially a rate comparison). As the q value is increased beyond zero, spike timing begins to impact the cost of matching the spike trains. In this way, q controls the *temporal resolution* of the spike train comparison. In the context of the SIMNETS algorithm, a high q parameter will bias the algorithm toward grouping neurons based on information encoded over fine timescales, whereas a low q parameter will bias the algorithm toward grouping neurons based on the information encoded over coarse timescales; the temporal accuracy of the algorithm can be characterized as 1/q. The VP method has the advantage of operating directly on point process data (preserving the natural statistical nature of spike trains), allowing for comparisons between relatively long spike trains (on the order of seconds) while preserving details of millisecond scale spike timing, by changing the cost assigned to shifting spikes in time (q parameter in the VP algorithm, see supplemental methods for details). Additionally, a point-process metric space can capture non-linearities in the neuron’s output space (Aronov and Victor, 2004; Fernandez and Farell, 2009).

In the current work, each single neuron SSIM matrix represents the relationship (i.e., similarity) between all spike trains for a single neuron for the specific time windows selected for all simultaneously recorded neurons. Figure 2. presents three examples of SSIM matrices derived from the spike trains of simulated neuron responses (Linear Non-Linear Poisson functional model; Meyer et al., 2016). Entries equal to zero (black) in the matrices represent identical spike trains (zero VP cost), while higher values represent increasing differences between the spike trains. For ease of viewing, the rows/columns of the matrices have been sorted according to simulated experimental conditions. Note that a specific ordering of the spike trains is not required for the subsequent comparisons (and is only included for illustrative purposes). It can be useful to graphically illustrate the relationship between individual spike trains across the experiment captured by the single neuron SSIM matrices using dimensionality reduction (Figure 2B). Here, we use PCA-initialized tSNE (Maaten and Hinton, 2008): a combined unsupervised dimensionality reduction technique that aims to preserve the local neighborhood structure, via t-SNE, as well as information related to the global shape, via PCA-initialization (Kobak and Linderman, 2021; Lee et al., 2015). The non-random PCA-initialization also ensures reproducibility across iterations (Kobak and Linderman, 2021). This mapping step is not necessary for the following steps of the SIMNETS analysis pipeline, but these maps can be useful to visualize and compare the features coded in the population. For example, in Figure 2B, the single neuron SSIM maps illustrate the separation of orientation selectivity in simulated neuron n1, the spatial frequency selectivity in n2, and the combined spatial and orientation selectivity of n3 (See Supplementary Figure S1; supplementary material for additional discussion and examples). Changes in the shape of the SSIM output space map may also indicate the influence of other variables which are not explicitly manipulated.

### 2.2 Computational similarity map: comparing single neuron output spaces

Each SSIM matrix characterizes the intrinsic geometry of the output space for a specific neuron. The next step of the algorithm compares these “computational fingerprints” across the full set of recorded neurons. This second-order comparison is made by correlating each pair of SSIM matrices (Figure 1, step 3; Figure 3). Here we use Pearson’s correlation (Figure 3A). Each correlation value is entered into a symmetrical (N x N) matrix (Figure 3B) representing the relationships across neurons, which we term the Computational Similarity (CS) matrix. Applying dimensionality reduction (here, PCA-initialized t-SNE; Maaten and Hinton, 2008) to the CS matrix creates a CS *map* (Figure 1, step 4; Figure 3C). Note that, while each colored point in a single neuron SSIM map represents an individual spike train (Figure 2C), each point in the CS map represents a neuron (Figure 3C). In the population CS maps, the distance between neurons is a relative measure of the similarity of computational fingerprints (the SSIM matrix). Therefore, neurons performing similar computations will tend to aggregate in clusters within the population CS map of neurons.

### 2.3 Identifying potential subnetworks: unsupervised cluster detection & validation procedure

After neurons are embedded in the CS map, the task of finding groups of neurons representing potential neuronal subnetworks can be addressed using a wide variety of clustering algorithms. Here, we use the k-means clustering algorithm, an efficient centroid-based clustering method, however, other density-based, distribution-based, or hierarchical-clustering methods would also be appropriate. The k-means algorithm works by iteratively assigning neurons into the pre-specified number of k clusters until the optimal solution is reached. However, the challenge is to ensure that the clustering structures identified by the k-means algorithm reflect genuine clusters within the CS neuron map and not false discoveries. To address this issue, we combined the k-means algorithm with two different cluster validation techniques, a silhouette graphical analysis and our novel shuffle-based statistical test, which together help avoid false cluster discovery (Figure 4).

Silhouette analysis is a validation technique used to determine the most likely number of clusters in a dataset (Figure 4; Rousseeuw, 1987). A silhouette value represents the ratio of the *between* to *within-cluster* distances: a point within an ideal cluster will be close to members of the same cluster and far from points assigned to different clusters, resulting in a high silhouette value. Choosing the cluster number with the highest average silhouette value—the Silhouette Coefficient (SC) score—maximizes the separation between potential clusters (Figure 4C, red point). We use a novel shuffled-based resampling procedure to assess the statistical significance of the SC score to avoid false cluster discovery. The test relies on creating 1000’s of surrogate datasets through a shuffling or randomization procedure that is applied to the rows/columns of the single neuron SSIM matrices We use the matrix permutation technique used in the Mantel test, which was specifically designed to randomize pairwise distance matrices. This is accomplished by permuting both rows and columns together in order to preserve matrix symmetry. In this way, it is possible to produce a new distance matrix for each neuron where the relationship between the trials (i.e., which trials are most similar) is randomized. This procedure effectively destroys any dependencies among the neuron’s single neuron SSIM matrices and, consequently, the associated clusters with the CS neuron map (Figure 4B). The empirical SC score is only considered statistically meaningful if it falls outside a distribution of surrogate SC values calculated from the 1000’s of surrogate datasets created in this manner (Figure 4C, see blue dots; for further insights on conditional resampling; see Amarasingham et al., 2012; Schemper, 1984). This unsupervised cluster detection and validation procedure supports the identification of statistically meaningful clusters of computationally similar neurons within the CS neuron maps.

## 3 Results

**Figure 5:**
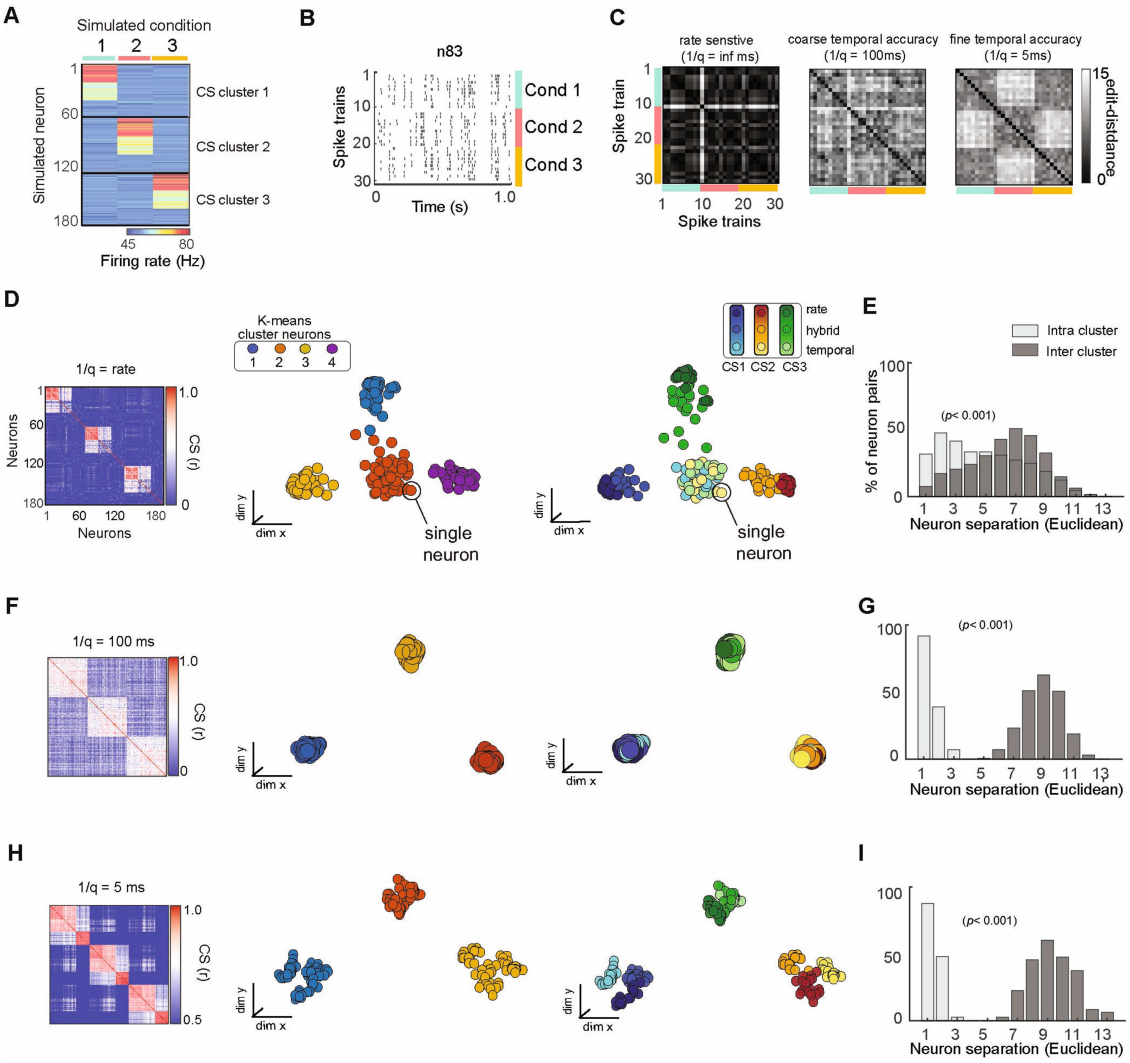
SIMNETS analysis framework successfully organizes a population of simulated neurons utilizing heterogeneous spike train encoding schemes into ground-truth subnetworks. **(A)** Population representation of spike trains from a population of 180 simulated neurons with firing rate constructed according to three different coding schemes (rate, temporal, mixed); 10 spike trains for each of three conditions (C1–3) for an ensemble of *N* = 180 simulated neurons. The neurons show a distinct pattern of spike rates across conditions, illustrating the different subnetwork groups. **(B)** Raster plot for one example neuron (n83) to illustrate a hybrid encoding scheme in which there is both rate change and different temporal structure in response to each of the conditions (C1-blue, C2-red, C3-yellow). **(C)** Three SSIM matrix representations for neuron #83, illustrating trial similarities organized according to stimulus blocks, illustrating the effect of q that emphasized firing rate (left; (1/q): infinite), coarse temporal (middle); 100 ms (coarse), and 5 ms (fine) temporal differences in spike pattern on each train. Note that the coarse and fine temporal matrices best reveal this neuron’s condition-dependent activity patterns, providing a quantifiable measure of similarity (distance) not readily available from the raster plot. **(D**,**F**,**H)** Population CS matrices for the population to identify subnetworks using pure rate codes (q = 0, no cost for shifting spikes in time), coarse temporal accuracy (1/q = 100 ms), or fine temporal accuracy (1/q = 5 ms). Neurons are colored according to clusters identified using k-means (left) or ground truth subnetwork designation (right). **(E**,**G**,**I)** Histograms showing normalized separation between neurons within each of the different SIMNETS maps for ground-truth “Intra-cluster” neuron pairs (light gray) and “Inter-cluster” pairs (dark grey).

**Figure 6:**
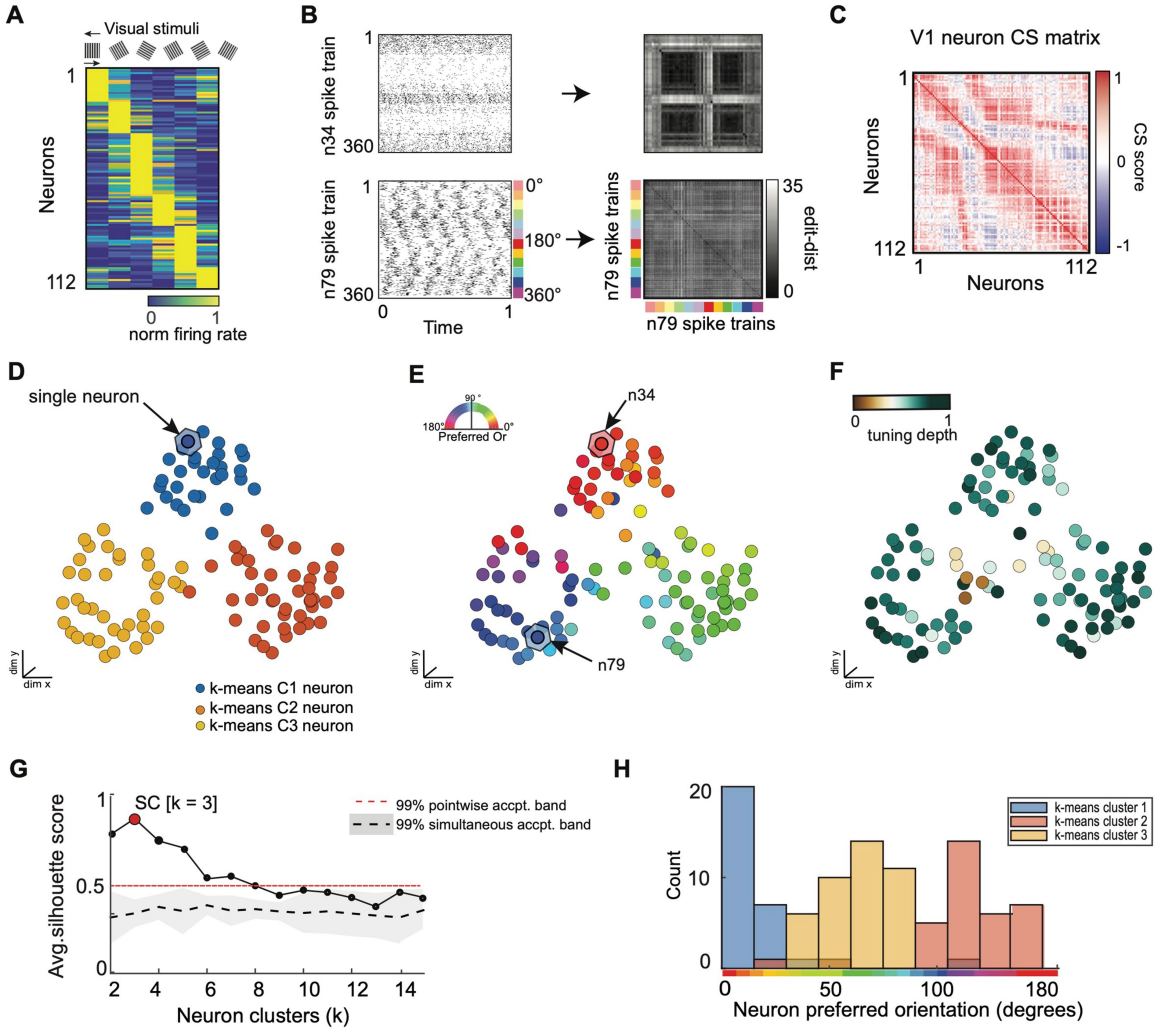
SIMNETS analysis framework captures known computational properties among a population of primate visual cortex neurons without the need for extrinsic labels or tuning models. **(A)** Normalized trial-averaged firing rates of a population of V1 neurons (*N* = 112, neurons) during the presentation of 12 different drifting grating stimuli (S = 360 spike trains) for 1.28 s at six different orientations: 0, 60, 90, 120, 150 degrees, and two drift directions (rightward and leftward drift). Neurons are ordered along the y-axis according to peak firing rate for visualization purposes, only. **(B)** Two example single neuron raster plots (n34 and n79) and their corresponding SxS single neuron SSIM matrices for VP (q = 35). The colored line indicates the stimulus orientation and drift direction. **(C)** SIMNETS NxN CS map. **(D–F)** Low-dimensional (3xd) population CS map, with neurons labeled according to k-means cluster assignments **(D)**, the neuron’s preferred stimulus orientation **(E)**, and their orientation tuning strength **(F)**. Example neurons, n34 and n79 **(B)**, are indicated with arrows in **(E)**. **(G)** Average silhouette score for the population CS map as a function of the number of clusters. Red circle indicates the SC at k = 3 (SC = 0.89; *p* < 0.0001; shuffled data). **(H)** Histogram showing the preferred orientation of the neurons within each of the three k-means CS neuron clusters from **(D)**.

**Figure 7:**
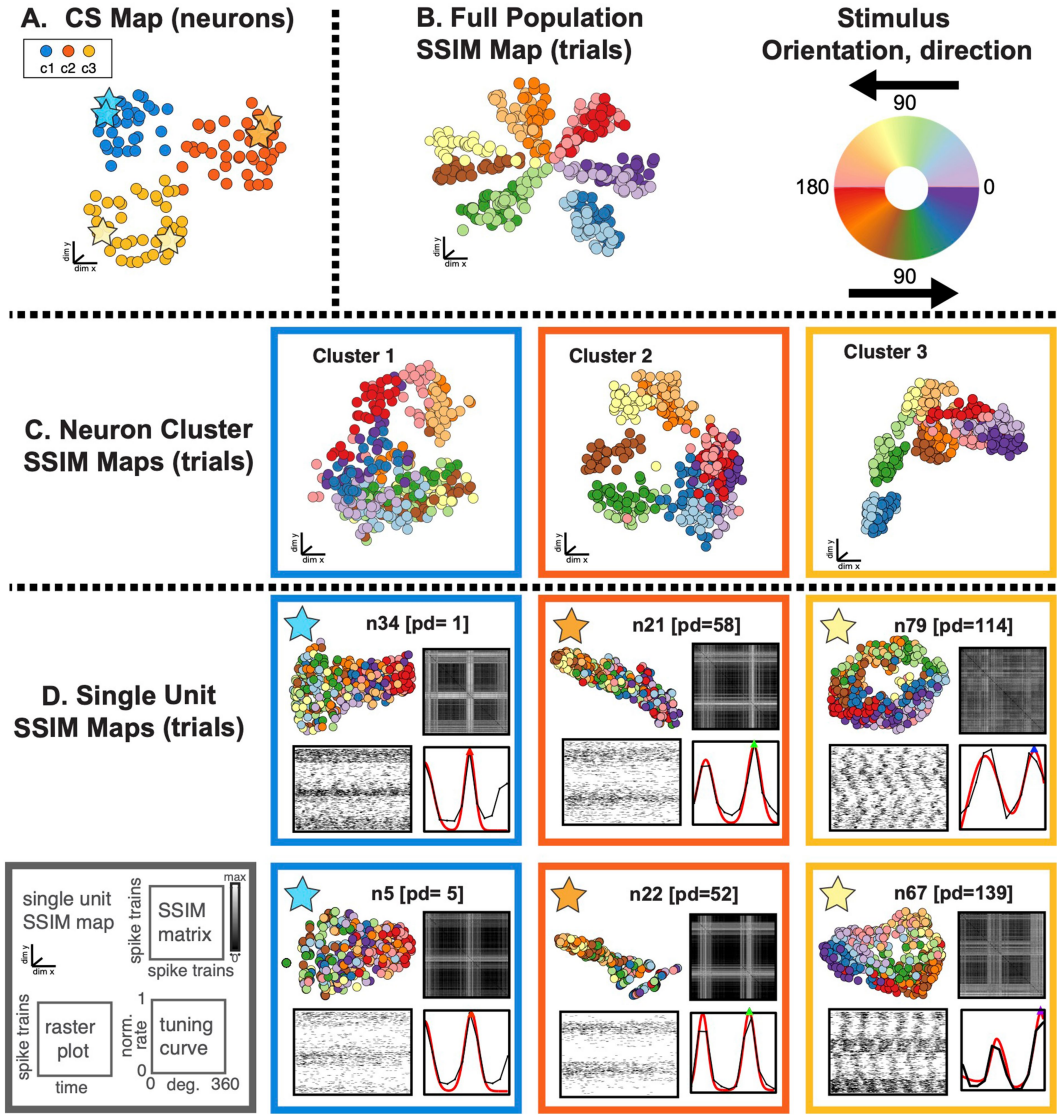
SIMNETS identifies clusters of primate V1 neurons with similar computational properties and displays the relationship between their activity patterns at multiple scales. **(A)** V1 SIMNETS CS neuron map: each point represents a neuron, and the distance between them reflects their computational similarity. Colors represent cluster assignment (c1–c3) obtained using k-means. The activity patterns for neurons highlighted with stars are shown in more detail on panel **(D)**. **(B)** Population-level SSIM map. Each point corresponds to a single trial; pairwise distances between points correspond to the dissimilarity among the V1 population spiking pattern on those trials. Colors correspond to the drifting grating orientation (Or); light vs. dark hues correspond to right (Or <180°) or left drift direction (Or >180°). **(C)** Mesoscale SSIM maps generated using subsets of neurons corresponding to the clusters shown in panel **(A)**. Note that the activity of each cluster of neurons reflects stimulus parameters in different ways, resulting in different SSIM map configurations. **(D)** Single unit SSIM maps, SSIM matrix, spike train raster plot, and parametric orientation tuning function (right drift direction in black, left in red) are shown for two example neurons from each of the three CS neuron clusters shown in panel **A**.

**Figure 8:**
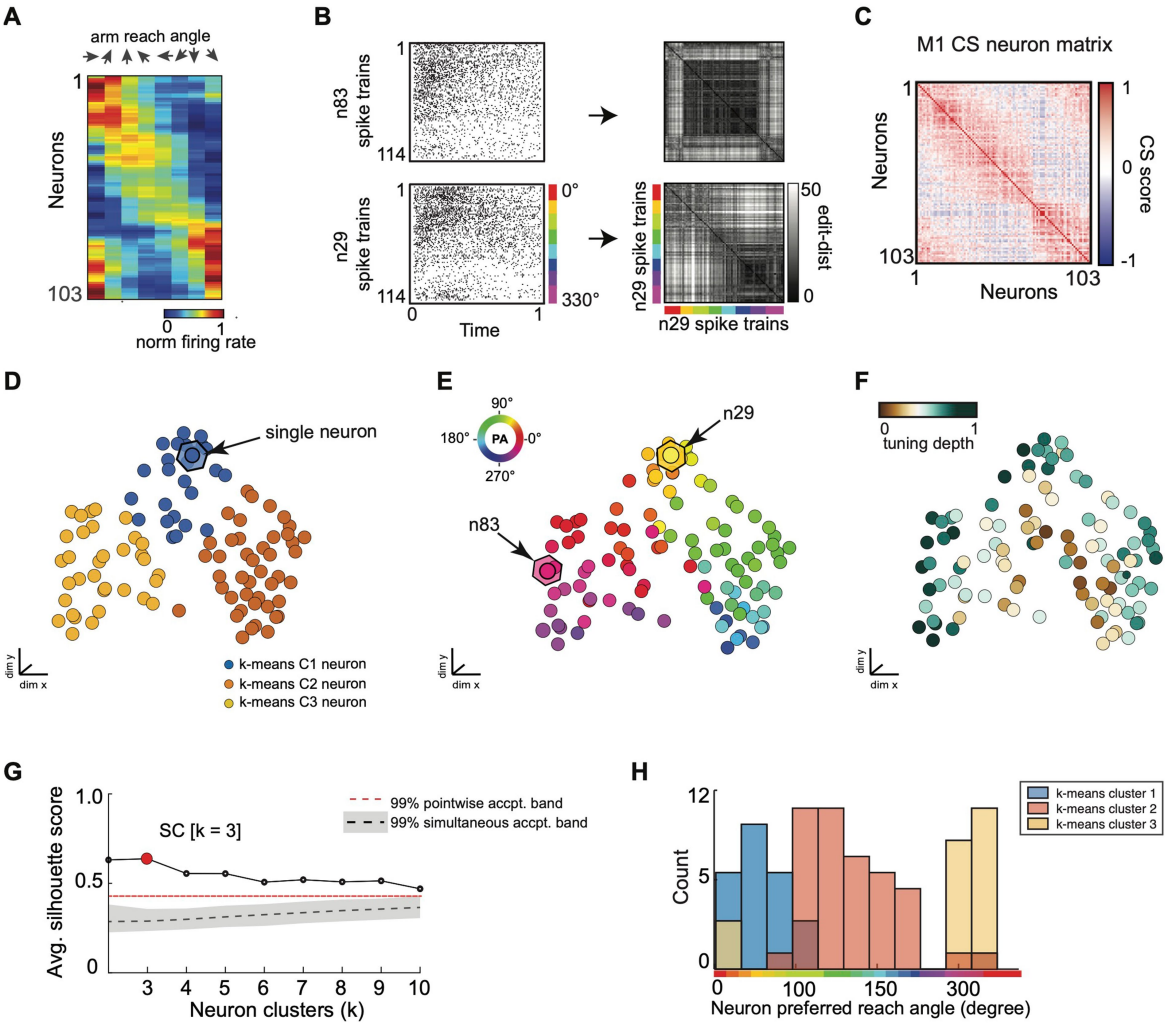
SIMNETS CS neuron map captures known computational properties among a population of primate motor cortex neurons without the need for extrinsic labels or tuning models. **(A)** normalized trial-averaged firing rates for a population of simultaneously recorded M1 neurons (*N* = 103, neurons) as a function of reach direction for a planar, 8-directional reaching task. For visualization purposes, neurons were ordered along the y-axis according to their peak condition-dependent firing rates. Neurons are ordered along the y-axis according to peak firing rate for visualization purposes, only. **(B)** Two example single neuron raster plots (n29 and n83) and their corresponding single neuron SSIM matrices for a VP edit-distance setting of q = 15 (i.e., 66 ms temporal precision). Colored line indicates the reach angle associated with each spike train. **(C)** SIMNETS NxN CS matrix. **(D–F)** Low-dimensional population CS map, with neurons labeled according to k-means cluster assignments **(D)**, the neuron’s preferred reach angle **(E)**, and tuning strength **(F)**. Example neurons, n29 and n83 **(B)**, are indicated with arrows in **(E)**. **(G)** Average silhouette score for the population CS map as a function of the number of clusters. Red circle indicates the SC at k = 3 (SC = 0.64; *p* < 0.0001; shuffled data). **(H)** Histogram showing the preferred reach angle of all neurons within each of the three k-means CS neuron clusters (shown in **D**).

**Figure 9:**
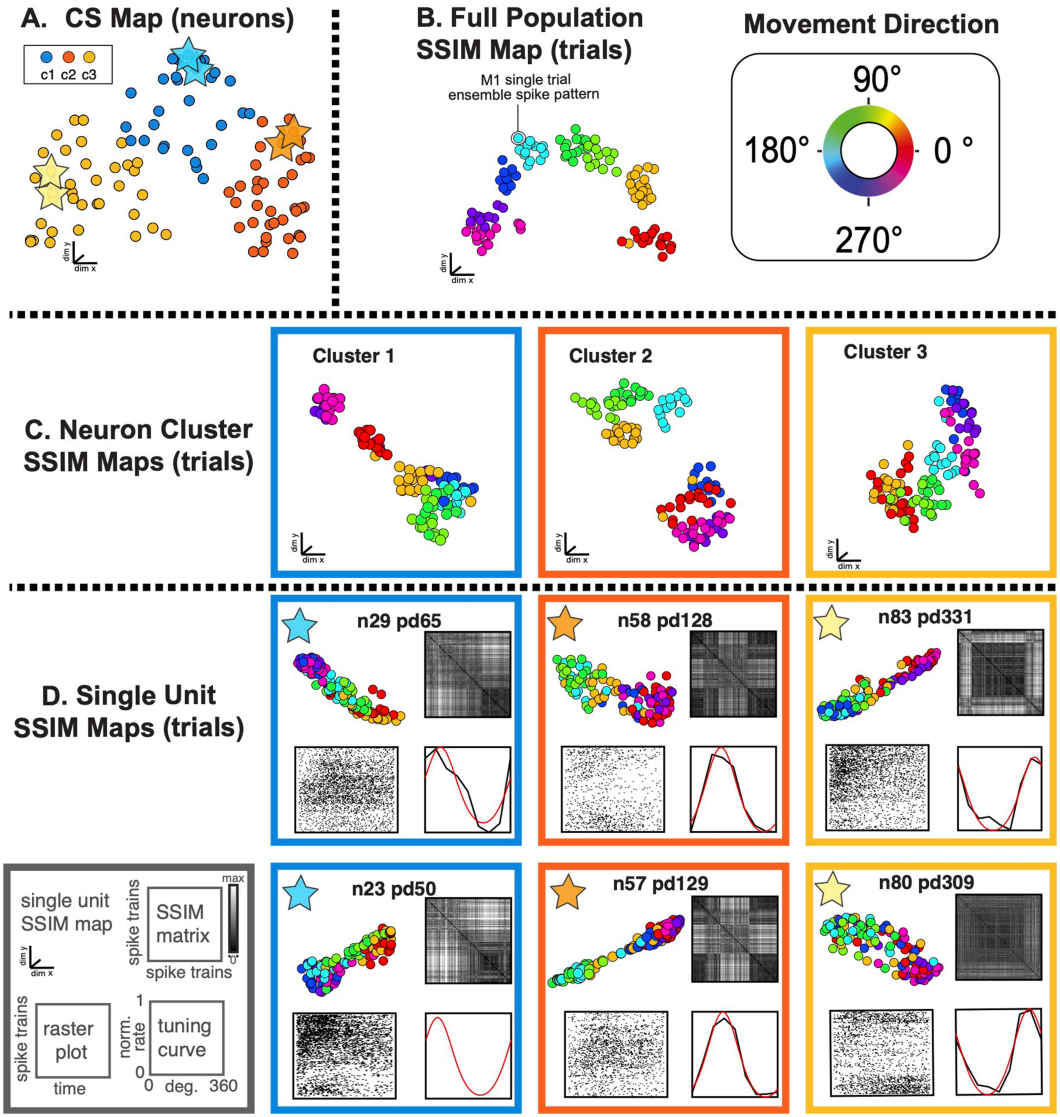
SIMNETS identifies clusters of primate motor cortex neurons with similar computational properties and displays the relationship between their activity patterns at multiple scales. **(A)** M1 SIMNETS CS neuron map: each point represents a neuron, and the distance between them reflects their computational similarity. Colors represent cluster assignment (c1–c3) obtained using k-means. The activity patterns for neurons highlighted with stars are shown in more detail on panel **D**. **(B)** Population-level SSIM map. Each point corresponds to a single trial; pairwise distances between points correspond to the dissimilarity among the M1 population spiking pattern on those trials. Colors correspond to trial condition labels (movement direction). **(C)** Mesoscale SSIM maps generated using subsets of neurons corresponding to the clusters shown in panel **A**. Note that the activity of each cluster of neurons reflects stimulus parameters in different ways, resulting in different SSIM map configurations. **(D)** Single unit SSIM maps, SSIM matrix, spike train raster plot, and parametric directional tuning function are shown for two example neurons from each of the three CS neuron clusters shown in panel **A**.

**Figure 10:**
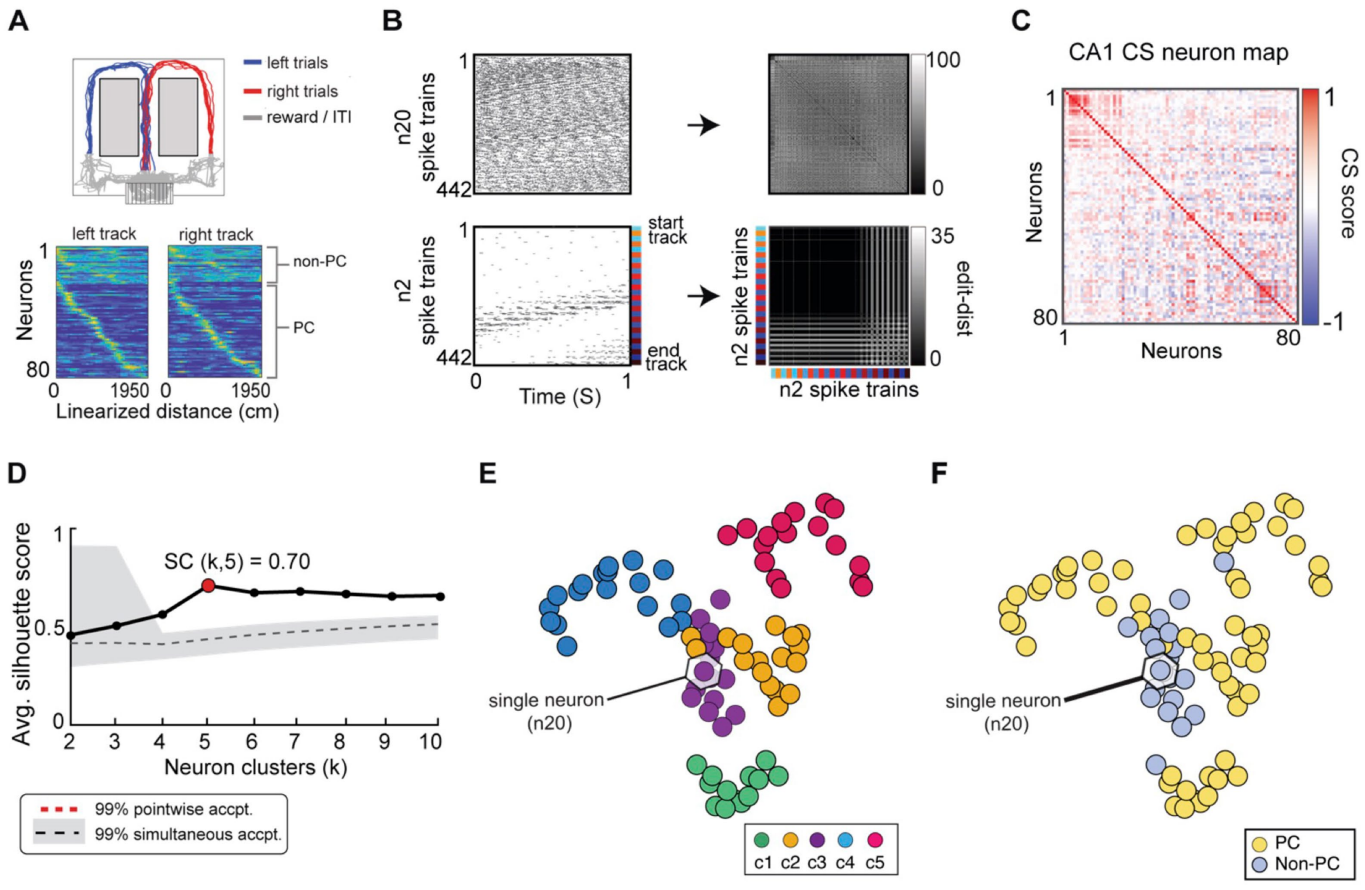
SIMNETS analysis framework captures the known computational relationships among a population of Hippocampal CA1 neurons in an unsupervised manner. **(A)** Task description: “*figure-8*” maze showing rat’s position during left–right alternation task (top). Red and blue lines show the rat’s location during correct right and left trials (T = 17, trials), respectively. Gray lines show the rat’s location during reward and inter-trial interval periods. Below: normalized firing rates are shown for each neuron (*N* = 80, neurons) as a function of linearized distance on track (50 cm bins) during left and right trials (below). For visualization purposes, neurons were ordered according to the latency of their peak response along the track and according to their characterization as a non-place cell (non-P. C, *N* = 20, light blue) or a place cell (P. C.; *N* = 60, yellow). **(B)** Two example single neuron raster plots (n7 and n20) showing S = 103 spike trains of 1-s duration (26 spike train segments per T trials) and their corresponding SxS single neuron SSIM matrices for VP [q = 35]. Colored line/dots indicate the rat’s location. **(C)** SIMNETS NxN CS matrix. **(D)** Average silhouette score for the population CS map as a function of the number of clusters. Red circle indicates the SC at k = 5 (SC = 70; *p* < 0.0001; shuffled data). **(E,F)** Low-dimensional (3xd) population CS map, with neurons labeled according to Ksc = 5 k-means cluster assignments **(E)**, and the neuron’s place-cell like firing properties **(F)** (P. C., blue dots; non-P. C., grey dots).

**Figure 11:**
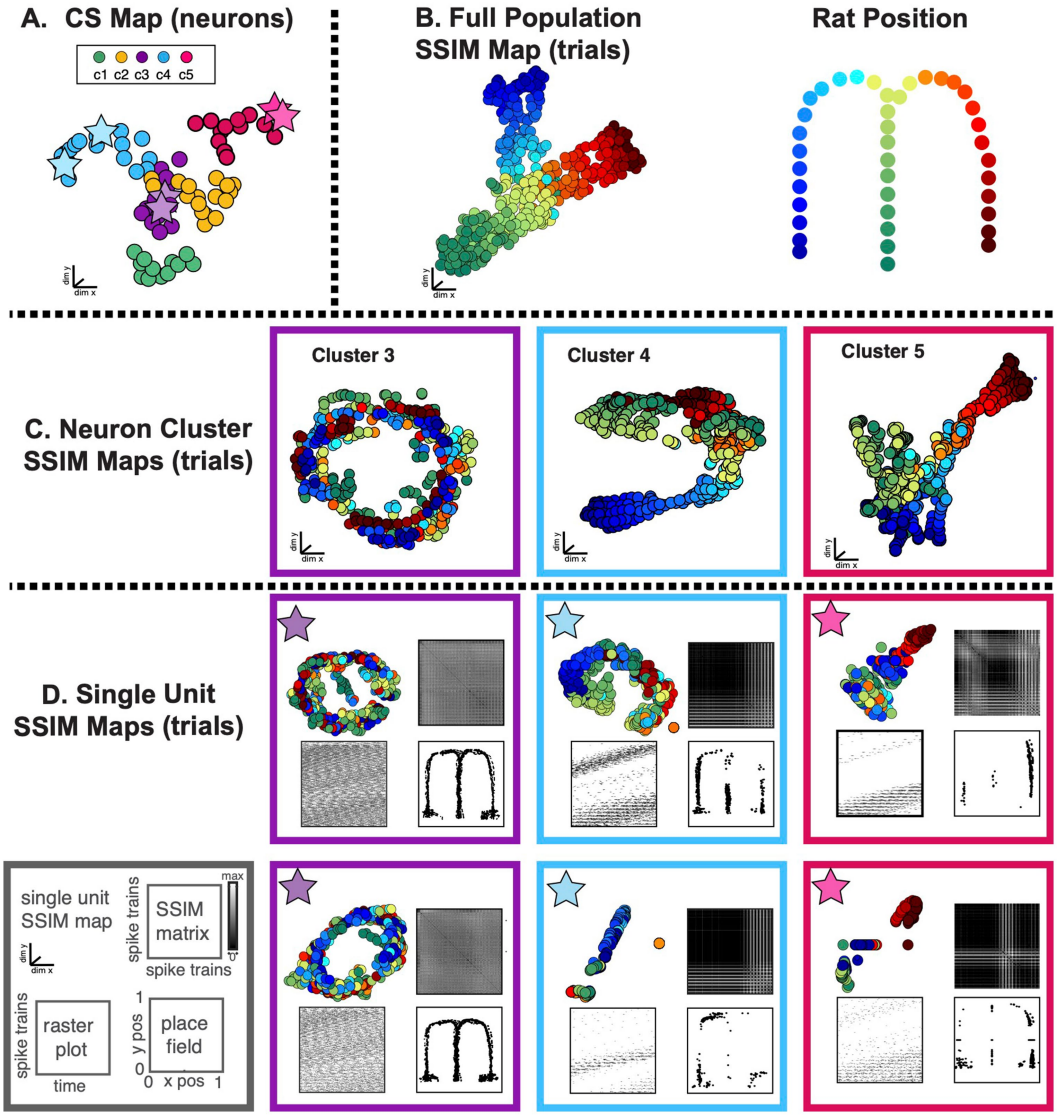
SIMNETS identifies clusters of hippocampal CA1 neurons with similar computational properties and displays the relationship between their activity patterns at multiple scales. **(A)** CA1 SIMNETS CS neuron map: each point represents a neuron, and the distance between them reflects their computational similarity. Colors represent cluster assignment (c1–c5) obtained using k-means. The activity patterns for neurons highlighted with stars are shown in more detail on panel **D**. **(B)** Population-level SSIM map. Each point corresponds to a single trial; pairwise distances between points correspond to the dissimilarity among the full CA1 population spiking pattern on those trials. Colors correspond to trial condition labels (movement direction). **(C)** Mesoscale SSIM maps generated using subsets of neurons corresponding to the clusters shown in panel **A**. Note that the activity of each cluster of neurons reflects stimulus parameters in different ways, resulting in different SSIM map configurations. **(D)** Single unit SSIM maps, SSIM matrix, spike train raster plot, and place fields are shown for two example neurons from three CS neuron clusters shown in panel **A**.

**Figure 12:**
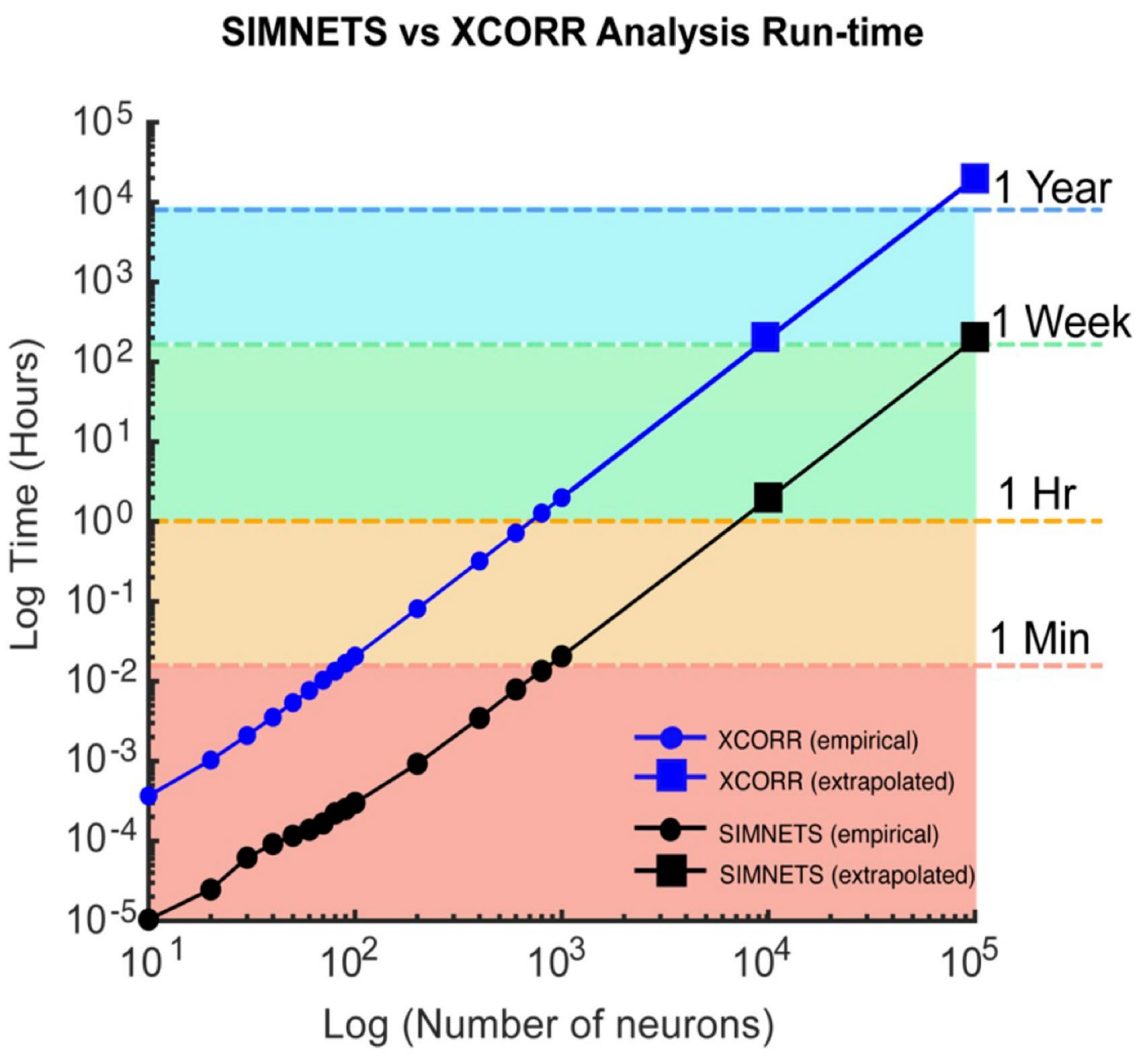
Computational run-time analysis for SIMNETS vs. cross-correlation algorithm. Log–Log plot showing SIMNETS algorithm (black lines) versus cross-correlation algorithm (XCORR, blue line) run-time as a function of neuron number (*N* = 10–100 k, neurons). Empirical data points (round) are the mean run-time across 15 iterations. Extrapolated data points (squares) were calculated from empirical data points for prohibitively large neuron population sizes (*N* = 10 k–100 k). Colored regions indicate whether the population size was analyzed in under 1 min (orange), 1 h, 1 week, or 1 year. Software was run through MATLAB on a standard personal computer: 6-Core Intel Core i7 processor, with 64GB of RAM. Cross-correlation analysis relied on MATLAB’s xcorr function.

We apply the SIMNETS analysis framework to four different datasets. First, we apply the algorithm to a population of simulated neurons for which spike patterns were constructed to simulate three subnetworks encoding information using firing rates, precise spike timing, or a combination of the two (Figure 5). Next, we apply SIMNETS analysis to three publicly available *in-vivo* experimental population recordings datasets from (1) macaque primary visual cortex (Kohn and Smith, 2016; Teeters and Sommer, 2009), (2) macaque primary motor cortex (Rao and Donoghue, 2014), and (3) rat CA1 hippocampal region field (Pastalkova et al., 2015; Wang et al., 2015) to illustrate the types of results the SIMNETS framework produces and how they may be leveraged for further exploratory analyses and hypothesis testing (Figures 6–11; for algorithm inputs/outputs for each dataset see: Supplementary Table S1). Finally, we highlight some of the positive attributes of the SIMNETS analysis framework and Toolbox that are particularly advantageous when analyzing large-scale neural recording data (Figure 12).

### 3.1 Simulated neuron population

We evaluated the performance of the SIMNETS algorithm using simulated neural data with known ground truth. This allowed us to verify that the algorithm clusters neurons with similar informational content even when they use different encoding schemes. We simulated a population of 180 neurons (N) composed of three computationally distinct subnetworks, SN1, SN2, and SN3 (Figure 5A). We generated an output structure for these subnetworks across three simulated experimental conditions (C1, C2, and C3). Each subnetwork emitted a similar “baseline pattern” for two of the conditions and a different pattern for a unique “preferred” condition (C1 for SN1, C2 for SN2, and C3 for SN3). Neurons from a common subnetwork were designed to encode similar information (i.e., respond differentially to one specific condition) using different encoding formats; for example, 20 neurons in SN1 encoded condition A through a change in spike rates (rate-coding), another set of 20 neurons in the same subnetwork responded with a change in the timing of their spikes (temporal-coding neurons), while the remaining neurons responded with changes in both spike rates and the spike time variations (mixed coding; e.g., Figure 5B). The other two subnetworks were also generated to include examples of rate coding, temporal coding, and mixed coding artificial neurons in the same proportions. We simulated 30, one-second-long spike trains for each of the 180 artificial neurons, which included 10 repetitions of each condition (S = 30 spike trains per neuron; See Supplementary Table S1 for analysis summary).

SIMNETS can be used to quantify spike train similarity and explore single unit encoding properties across an experiment without assuming what coding schemes are relevant or what variables are generating spike train differences. It is also possible to examine the temporal accuracy of single unit spiking. To illustrate this point, the SIMNETS algorithm was applied using three different temporal accuracy values for the VP spike train similarity metric: a pure rate code (q = 0, i.e., no added cost for shifting spikes in time), 100 ms (q = 10), 5 ms (q = 200). The q parameter determines how sensitive the similarity metric is to differences in the timing of individual spikes, by setting an upper limit on how far spikes can be shifted in time (See Figure 5C, for three different single neuron SSIM matrices calculated using different q values). With a setting of q = 0, the simulated neurons operating with a rate-based encoding scheme and mixed coding neurons are grouped into three functionally distinct clusters in the CS map (Figure 5D, 2nd column), while temporal coding neurons form a single cluster at the center of the map (Figure 5D, 3^rd^column). As the value of q increases, the VP algorithm “cost” function becomes sensitive to differences in spike timing in addition to the total number of spikes (Victor and Purpura, 1996). SIMNETS correctly groups all neurons into three distinct clusters that reflect the ground-truth functional ensemble assignments (Figure 5F). At the highest q value (Figure 5H), the CS map shows sub-groupings within each of the three ground-truth subnetworks that correspond to neuron’s encoding formats (Figure 5H, 3^rd^ column); however, the optimal number of clusters remains in agreement with the ground-truth functional subnetwork assignments (K_sc_ = 3). By specifying a higher partition value for the k-means clustering step of the algorithm (e.g., k = 9), the sub-groupings within the detected clusters are defined by the coding properties of the neurons. For a demonstration of the interaction between the cluster number and the SIMNETS hyperparameters, perplexity and q, see Supplementary Figure S2.

To evaluate whether SIMNETS could successfully identify subnetworks, we compared the distribution of Euclidean distance between individual neurons in the SIMNETS CS map for neuron pairs within and between the artificially generated (‘ground truth’) subnetworks (Figures 5E,G,I, histogram). The within-subnetwork similarity was significantly higher than between-subnetwork values in all cases (Mann–Whitney *p* < 0.001). For q values > 0, there was no overlap between the two distributions, indicating the complete separation of the artificially generated subnetworks. Our results demonstrate that the SIMNETS algorithm can accurately separate neurons according to their computational properties (the latent variables upon which they operate), even if they employ different coding schemes to represent information using spike rates or temporal patterns.

### 3.2 Application to empirical multi-neuronal recordings

We next applied SIMNETS to three distinct datasets of simultaneously-recorded, multiple single-unit recordings from three different areas in two species: (1) nonhuman primate (NHP) primary visual cortex (V1) during visual stimulation (Kohn and Smith, 2016; Smith and Kohn, 2008), (2) NHP motor cortex (M1) neural recordings during a center-out reaching task (Rao and Donoghue, 2014), and (3) rat CA1 hippocampal region recordings during a left–right alternation maze task (Pastalkova et al., 2015; Pastalkova et al., 2008). These datasets were chosen because the functional roles of the neurons in these areas have been reasonably well-established and the information content of the single neuron and ensemble spiking patterns extensively described (Gur and Snodderly, 2007; Livingstone and Hubel, 1984; Rao and Donoghue, 2014; Smith and Kohn, 2008; Truccolo et al., 2008).

We note that these results are meant to illustrate the capabilities and utilities of the SIMNETS analysis pipeline and are not intended to represent a full characterization of the role of any identified putative subnetworks. For a full analysis and interpretation, many more datasets (sessions/subjects) would be required, as would various parameter sweeps to evaluate computational processes.

### 3.3 Macaque V1 neural population recordings during visual stimulation

We analyzed a dataset of 112 simultaneously recorded V1 neurons (recorded using a 96-channel “Utah” electrode array) during the presentation of drifting sinusoidal gratings in an anesthetized Macaque monkey (Kohn and Smith, 2016; Figure 6A). We extracted 1 s of spiking data from the first 30 repetitions of each stimulus (*S* = 360), starting 0.28 s after stimulus onset (see Figure 6B for raster plots and single neuron SSIM matrices for two example neurons) to capture the neural stimulus response period (eliminating response delay). SIMNETS was applied to these NxS spike trains generated with a fixed temporal accuracy setting of 1/*q* = 50 ms. The resulting NxN CS neuron matrix is projected into a lower-dimensional CS space upon which further statistical and clustering analyses are performed (Figures 6D–F). An unsupervised k-means cluster analysis identified 3 clusters of related single neurons in the V1 CS map (Figures 6D,G; SC = 0.78, peak max average silhouette value), indicating that computational features did not fall along a functional continuum but instead were organized into three functionally distinct putative subnetworks.

To validate the performance of the SIMNETS algorithm, we examined the relationship between the structure of the CS map and the known tuning properties of V1 neurons. We used classic parametric tuning models to estimate the receptive field properties of all neurons and labeled the neurons in the CS neuron map according to their “preferred” stimulus orientation (Figure 6E) and tuning depth (Figure 6F). Each neuron’s preferred orientation was calculated by fitting a Gaussian distribution to the stimulus-dependent firing rates and finding the orientation that maximizes the function over the range of orientations angles (*θ* = [0,180]) across both left and right drift directions. Tuning depth was calculated as the normalized difference between the peak and trough of the tuning function (See Supplementary methods). Again, we emphasize that the SIMNETS procedure is an entirely unsupervised approach and does not rely on any knowledge of the experimentally-induced neuron tuning functions to generate the CS map or to identify putative CS neuron clusters. We simply labeled each neuron with values obtained from its relationship to an experimental variable (here, grating direction) *after* the neurons were plotted in the SIMNETS CS map. A pairwise circular-linear correlation (*r*_cl_) statistical analysis revealed a strong positive correlation between neurons’ distance in the low-dimensional CS neuron map and the differences in their preferred orientation parameters (Pearson, *r*_cl_ = 0.89). This relationship confirmed that neurons with similar preferred orientation were generally mapped near one another in the CS map (Figure 6E). The exception to this trend was observed for neurons with weaker tuning strengths (Figure 6F, brown points), which aggregated at the center of the CS neuron map. Overall, the distribution of the neurons’ preferred orientations corresponded well with the three observed clusters in the CS neuron space (Figure 6H). Collectively, these results confirm that the neurons’ sensitivity to the different stimulus orientations is a major contributor to the structure of V1 computational architecture. The CS map also reveals the presence of three computationally distinct groups of neurons within the context of this experimental paradigm.

The overall agreement between known V1 tuning features and the CS map generated using SIMNETS suggests that the algorithm will be able to identify computational architectures for neural systems where the tuning functions are not known *a priori*.

The DR techniques used to visualize the computational geometry representations of the single neuron SSIM maps (introduced in Figure 2) can be expanded to represent the combined output space of multiple neurons simultaneously, such as the CS neuron clusters (i.e., putative subnetworks) or the full neuron population. For multi-neuron SSIM maps, each point corresponds to the collective spiking pattern generated by all neurons within an ensemble (e.g., CS neuron cluster or full population) on a single trial, and the distance between points corresponds to the difference (spike train distance) in the neurons collective spike pattern across time. Here, we use this approach to visualize the relationship between activity patterns for the full neuron ensemble (Figure 7B), the three CS neuron clusters (Figure 7C), and example single neurons from each of the three CS neuron clusters (Figure 7D). Standard single neuron plots (parametric tuning functions and rasters) were also included for comparison.

### 3.4 Macaque M1 neural population recordings center-out reaching task

As a second example, we applied the SIMNETS algorithm to a dataset of 103 M1 neurons recorded using a 96-channel electrode array in a macaque performing a planar, 8-direction arm reaching task (see Methods for more details). We extracted 1-s long spike trains (S = 114) from each neuron during all trials where the monkey successfully reached the cued target, starting 0.1 s before movement onset (Figure 8A; see Supplementary Table S1 for algorithm inputs and parameters). SIMNETS was applied to these NxS spike trains generated with a fixed temporal accuracy setting of 1/*q* = 50 ms. SIMNETS analysis revealed that the neurons were organized into three neuron clusters in the M1 CS map (Figures 8D,G, SC score = 0.71, M1 peak average silhouette).

To examine the relationship between these clusters and functional properties of the neurons, we characterized each neuron’s preferred reach direction (PD) angle and tuning depth (Figure 8F) in a similar manner to the V1 dataset. Each neuron’s PD was estimated by fitting a von Mises distribution to the firing rates as a function of direction (Supplementary Figure S3; Mardia and Zemroch, 1975) A circular-linear correlation (rcl) analysis between the difference in PD and CS map distance between pairs of neurons revealed a significant positive relationship (Pearson, rcl = 0.92; *p* = 0.001), confirming that neurons with similar preferred directions tended to cluster together in the CS map, while neurons with dissimilar preferred directions were spatially distant (Figures 8D,E, right plot). After plotting the distribution of preferred reach direction, we again observed that the neuron tuning properties were similar across each of the three detected CS neuron clusters (Figure 8H).

SIMNETS analysis suggests that the computational properties of M1 neurons are not uniformly distributed along a functional continuum, as evidenced by the distinct CS neuron clusters identified in the population CS space. The separation between the CS neuron clusters is most apparent for CS neuron cluster 2 and cluster 3 (Figure 8D, orange and yellow CS clusters), which mirrors the discontinuity in the distribution of preferred reach angles (Figure 8E). These results agree with previous findings, which support the hypothesis that the biomechanical constraints of the limb are reflected in an uneven distribution of preferred directions among motor cortical neurons (Lillicrap and Scott, 2013).

As with the V1 dataset, we used a multi-scale latent space analysis to visualize the relationship between spiking patterns across different scales within the CS map (Figure 9A), from the full ensemble (Figure 9B) to CS neuron clusters indicative of potential subnetworks (Figure 9C) and individual neurons functional properties (Figure 9D). Standard single neuron plots (parametric tuning functions and raster plots) are again included for comparison (Figure 9C). Each M1 neuron had a unique single neuron SSIM map that captured several computationally relevant features of the neurons’ spike train outputs (Figure 9C; SSIM matrices/maps for two example neurons). In contrast to the regular conical and circular parabolic shapes of the V1 single neuron SSIM spaces (Figure 7C), the M1 SSIM maps displayed more irregular and heterogenous global geometries (Figure 9D).

### 3.5 SIMNETS subnetworks in a population of rat hippocampal CA1 neurons

Lastly, we assessed the CS structure of a population of hippocampal neurons using SIMNETS, a region that is both functionally and structurally quite different from the primary visual and motor neocortical areas examined above. We applied SIMNETS to a publicly available dataset of rat CA1 hippocampal neurons recorded using multi-site silicon probes in a rat performing a left/right-alternation navigation task in a “*figure*-8” maze (Pastalkova et al., 2015; Figure 10A). All neurons with firing rates greater than 5 Hz were included in the analysis (*N* = 80/106 recorded neurons). The rat performed 17 correct trials (T = 17, trials), taking an average of 4.3 s to reach the reward location at the end of the left or right arm of the maze. Spike trains used for the SIMNETS algorithm were obtained by extracting 1-s of spiking activity for each 80 mm increment of distance traveled by the rat (Figure 10B, *left*, example single neuron raster plots with S spike trains; see Supplementary methods). This resulted in a set of S = 442 spike trains (17 trials x 26 time-epochs) per neuron.

SIMNETS was applied to the set of SxN neurons, using the VP temporal sensitivity setting of q = 35 (Figures 10B,C; see Supplementary Table S1 for all algorithm inputs/outputs). After generating the low-dimensional CA1 population CS neuron map, we characterized the organizing properties of the neuronal relationships using the unsupervised k-mean cluster detection/validation procedure. This procedure identified Ksc = 5 statistically significant CS neuron clusters within the CA1 CS neuron map (SC = 0.70, peak max average silhouette value; Figure 10D), suggesting that the neurons are organized into five computationally distinct clusters of neurons. To verify this, we used two different classic functional characterization approaches to assign functional ids to the neurons within the SIMNETS CS map and assess their functional organization within the context of the CS neuron map (Supplementary Figure 10F). Specifically, we categorized the CA1 neurons as having place cell-like activity (*n* = 60, place-cells, PCs) or lacking place cell-like activity (*n* = 20, non-place-cells, non-PCs) using an information-theoretic measure (Skaggs et al., 1992) and a spatial firing rate tuning functions (Figure 10B, example single neuron raster plots; see Supplementary methods for analysis details). As is reported in other studies, some of the neurons categorized as place-cells exhibited complex firing responses, such as multiple peak place-dependent firing responses (Supplementary Figure S3D, show example single neuron response functions). An examination of the physiological properties of the identified k-means neuron clusters revealed that one cluster was almost entirely (95%) composed of non-PCs (Figures 10E,F, K-means cluster c3, purple), while the other four k-means CS neuron clusters were entirely or almost entirely (>92%) composed of PC’s (Figures 10E,F). This indicated one of at least three possibilities: (1) the “non-PC CS cluster,” cluster c3 corresponded to a cluster of untuned, “uninformative” neurons, (2) that the individual neurons computed a common task-related signal that was unrelated to place-coding, or (3) that place-coding signals were only apparent at the level of the neuronal cluster rather than the individual neurons (Figure 10E, cluster c3). While a full exploration of these different possibilities is beyond the scope of this work, we do provide a demonstration of how the SIMNETS CS neuron maps may be used to visualize the relationship between spiking patterns across scales for hypothesis formation and testing (Figure 11).

Once again, we used a multi-scale latent space analysis to visualize the relationship between spiking patterns across different scales within the CS map (Figure 11A), from the full ensemble (Figure 11B) to CS neuron clusters indicative of potential subnetworks (Figure 11C) and individual neurons functional properties (Figure 11D). As expected, the spatial layout of the task-space was reflected in the topology of the population-level SSIMS map (Figure 11B), while each cluster-level SSIMS maps captured the place-dependent modulation of the clusters spiking patterns as the rat traversed a specific region of the maze (Figure 11C). Surprisingly, the “non-PC neuron cluster,” (Figure 11C, cluster c3) did not appear to reflect a cluster of non-responsive or “uninformative” neurons; instead, it had a cylindrical shape that resulted from at least two dominant patterns of spike pattern modulation: a within-trial periodic spiking pattern in the x-y plane (Figure 11B, cluster c3, x-y viewing angle) and a location or distance-dependent variance along the z-axis (See Supplementary Figure S3C, for x-z viewing angle). This cylindrical shape was also observed in the single neuron SSIM maps of the neurons within cluster c3 (see Figure 11C, purple box/stars, neurons c3-n7 and c3-n20), however, the distance-dependent x-z variance was less apparent at the single neuron level. Overall, this suggests that the activity of the non-PC neuron cluster, c3, displays dynamics that may reflect a different task variable that is in some way correlated with the rat’s performance on the task (e.g., head direction, speed profile along maze arms) or, alternatively, the intrinsic circuit dynamics (Pastalkova et al., 2008; Richard et al., 2013; Wang et al., 2015). Latent spaces displaying 3-dimensional ring topology reflecting animal position and running direction encoding have been recently described in CA1 (Esparza et al., 2025). Further investigation of this phenomenon is outside of the scope of the present work but is an example of how SIMNETS may be used to quickly uncover different types of functional relationships among neural representations that might not be otherwise evident when using classic population and single unit analyses. These results highlight the advantages of applying SIMNETS to neural recordings where tuning properties or other coded variables are not readily apparent or unknown *a priori*.

### 3.6 Computational efficiency and analysis run-time

The SIMNETS framework can rapidly analyze large numbers of neurons using a standard personal computer (e.g., 6-Core Intel Core i7, 64GB of RAM). With the current Matlab® implementation (see included SIMNETS software package), the simulated neuron population (*N* = 180, S = 30) and Macaque M1 neuron population (*N* = 103, S = 360) were processed in less than 2 s. Larger datasets, such as the Macaque V1 neuron population (*N* = 112; S = 360) and rat hippocampal CA1 neuron population (*N* = 80, S = 442), take approximately 10 s to process. This is significantly faster than a typical cross-correlation-based functional connectivity analysis. For example, a dataset of 1,000 neurons can take an hour to processes with a standard cross-correlation analysis (Figure 12, blue line; utilzing Matlab® xcorr function), whereas our implementation of the SIMNETS algorithm takes only about a minute to process the same dataset (Figure 12, black line). The SIMNETS analysis is faster because it involves fewer spike train comparison operations (NxSxS) than the cross-correlations analysis (N2xSxS, across all time-lags), and the number of spike train comparisons per added neuron has a favorable linear scaling. If parallel or distributed computing resources are used to calculate the single neuron SSIM matrices, the execution time can be reduced even further to seconds or milliseconds. This would be especially useful for datasets with larger trial numbers (e.g., S > 1,000), which are more computationally intensive than datasets with larger neuron numbers.

## 4 Discussion

Advances in multi-electrode recording technology have made it possible to record or image the activity of thousands of individual neurons simultaneously (Chung et al., 2022; Hsieh and Zheng, 2019; Paulk et al., 2022; Steinmetz et al., 2021; Vogt, 2019). The shift in focus from studying single neurons to population levels responses has brought about a new era of discovery, innovation, and collaboration (Buzsáki, 2004; Cunningham and Yu, 2014; Hurwitz et al., 2021; Kohn et al., 2020; Paninski and Cunningham, 2018; Paulk et al., 2022; Saxena and Cunningham, 2019; Urai et al., 2022); highlighting the need for new theoretical and analytical frameworks that can bridge the “representational gap” between neuron-level and population-level computation.

With this problem in mind, we devised SIMNETS, a novel relational analysis framework. SIMNETS was designed to generate low-dimensional maps of computational similarity across populations of simultaneously recorded neurons. The critical difference between SIMNETS and traditional functional connectivity measures is that we assess similarities among the neurons’ intrinsic latent space geometries, rather than the similarities of the neurons’ response time course (e.g., correlation between firing rates). We emphasize that in contrast to neuronal functional connectivity analyses that measure time series correlations among the firing rates vectors or spiking patterns between *different* neurons (Abeles and Gat, 2001; Aertsen and Gerstein, 1985; Gerstein and Michalski, 1981; Grün et al., 2002b; Humphries, 2011; Kiani et al., 2015; Stuart et al., 2002) SIMNETS only directly compares spike trains generated by the same neuron, i.e., self-similarity. We performed spike train comparisons using the VP metric, a point process edit distance measure that can be tested over a variety of temporal accuracy settings. By quantifying the similarity of a neuron’s spike train outputs across all trials (a within-neuron 1^st^-order comparison), we generate a geometric representation of each neuron’s spike train output space, which we use as a *“computational fingerprint*” which is captured in its spike train similarity matrix (Figure 2). This approach takes into account changes in spiking related to all the variables in the experiment (controlled, uncontrolled, and noise) in an unsupervised manner, that is, without the use of trial labels or neuron functional labels (Kohn and Smith, 2016; Pastalkova et al., 2015; Rao and Donoghue, 2014). SIMNETS projects the neuron population into a common coordinate space that captures the relationships between the neuron’s computational fingerprints. The distance between neurons in the CS space provides a quantifiable measure of computational similarity, and CS neuron clusters reveal potential subnetworks with distinct computational properties. We used a dimensionality reduction method that aims to preserve both local and global structure (e.g., PCA initialized t-SNE; Maaten and Hinton, 2008; Kobak and Linderman, 2021; Lee et al., 2015). The structure of the CS maps can provide additional insight into the type of information that is being computed potential and subnetwork organization. SIMNETS could help resolve long debated issues as to whether classes of neurons that represent a unique set of features belong to a discrete computing module, or alternatively, whether there exists a functional gradient of computational properties.

We see the novelty of the SIMNETS framework as combining similarity metrics, dimensionality reduction and clustering to organize neurons into functional groups. The specific combination of algorithms chosen for the implementation of SIMNETS presented here were selected because they have been thoroughly validated (on an individual basis) through previous work. However, it is important to note that each of these steps can be accomplished using a variety of methods and could potentially benefit from recent developments in each of these areas. Other analysis pipelines could also benefit from employing SIMNETS as a preprocessing step. For example, applying DR techniques to examine the low dimensional latent spaces associated with clusters in the SIMNETS CS map provides another way to probe the potential computational properties of putative subnetworks (Figures 7, 9, 11). This strategy could take advantage of recent advances in DR methods that incorporate tensor analysis (slice TCA, Pellegrino et al., 2024), ensemble dynamics (NoMAD, Karpowicz et al., 2025), or explicitly include task variables to enhance low dimensional representations (DAD, Dyer et al., 2017; CEBRA, Schneider et al., 2022).

Finally, the combination of a short processing time (< 1 min per 1,000 neurons), a computational complexity that scales near-linearly with the size of the neuron population (Figure 12), and ease of parallelization, makes SIMNETS an efficient tool for exploring very large-scale neuron populations with readily available hardware.

### 4.1 Algorithm validation

We tested and validated SIMNETS using simulated data with known ground truth as well as three different experimental neural recording test datasets to demonstrate the general applicability of the analysis framework across a range of coding schemes, neural systems (visual, motor, cognitive), behaviors (awake or anesthetized), and different species (rat and macaque). This included a demonstration of a novel shuffle-based statistical procedure based on the Mantel test for identifying non-spurious CS neuron clusters within a low-dimensional population CS neuron map.

Our analysis of simulated data with ground-truth subnetworks and known single neuron properties (i.e., encoded content and encoding timescale) demonstrates the current implementation of the SIMNETS algorithm can capture a neuron’s local and global computational relationships, even under conditions where computationally similar neurons relied on very different encoding schemes (e.g., rate coding vs. fine temporal coding schemes). This agnosticism can be beneficial when analyzing neurons that generate highly heterogeneous spike train outputs because of differences in their internal machinery (e.g., inhibitory vs. excitatory neurons) yet still reflect task-relevant information (Churchland and Shenoy, 2007; Diba et al., 2014; Martina et al., 1998). We demonstrated how the VP metric temporal accuracy parameter (q) could be used to interrogate the effect of temporal resolution on the computational configuration of the neurons.

The application of SIMNETS to three publicly available multi-neuron recording datasets validated the capabilities of the method in revealing known dominant feature representation in V1, M1, and CA1 (orientation, direction, place, respectively) without imposing stimulus or movement-driven parametric tuning models *a priori.* Of note, the results show that the computational properties of neurons are not uniformly distributed, but instead are organized into apparent subnetworks (i.e., CS clusters in the low-dimensional CS map) that emphasize certain collections of feature representations. Our results also suggest that SIMNETS may be able to detect distinct subnetwork structures hypothesized to support ensemble place-coding or complex feature conjunctions (Buzsáki, 2019; Diba et al., 2014). Although it was beyond the scope of this report to demonstrate the functional or computational significance of the detected putative subnetworks, our results suggest that the detected clusters are physiologically meaningful; our subsequent unsupervised cluster validation statistical procedure further provided support to these findings. Finally, our choice of datasets allowed us to demonstrate that this method generalizes well to neural recordings from a variety of brain regions, including sensory areas (Kohn and Smith, 2016), motor areas (Rao and Donoghue, 2014), and memory/cognitive areas (Pastalkova et al., 2008); different species, including rat and non-human primate; and across different recording technologies including laminar silicone probes (Buzsáki, 2004) and fixed-dept multi-electrode arrays (Maynard et al., 1997).

Collectively, our results demonstrate that single neuron SSIM matrices provide a simple, scalable, and powerful format for characterizing the computational properties of neurons, as well as identifying computational similarities and differences between them. In addition, examining the arrangement of neurons in the CS maps may suggest novel coding schemes that are yet unexplored by experimenters, generating new hypotheses for future experiments.

### 4.2 Comparison to existing approaches

The SIMNETS analysis framework builds upon rich theoretical and mathematical literature spanning multiple domains and disciplines. Geometric and metric space representational models of similarity have a long history of application in the field of psychology where they have been used to model the perceptual relationships between sensory stimuli as latent or low-dimensional perceptual metric-space (Forstmann, 2011; Goldstone et al., 1991; Hyman, 1974; Shepard and Chipman, 1970; Shepard, 1987; Shepard, 1964; Thurstone, 2017; Tversky, 1977; Zenker and Gärdenfors, 2015). This approach has recently been adapted to study the intrinsic structure of neural representational spaces in visual, motor, and cognitive brain regions (for reviews, see; Edelman et al., 1998; Haxby, 2012; Kriegeskorte et al., 2008; Kriegeskorte and Kievit, 2013; Lehky et al., 2013). Several mathematical variations of this framework have been developed to study the information content of macroscale fMRI BOLD signals (Diedrichsen and Kriegeskorte, 2017; Haxby et al., 2001; Kriegeskorte and Bandettini, 2007), and both population-level and neuron-level spiking patterns (Houghton and Victor, 2010; Kiani et al., 2007; Vargas-Irwin et al., 2015a, 2015b; Victor and Purpura, 1996). To the best of our knowledge, this is the first work that uses a measure of 2nd-order point-process similarity analysis to generate latent space embedding of a collection of individual neurons.

The concept of a low-dimensional embedding that captures the functional relationship between spiking neurons was introduced in the seminal papers by (Baker and Gerstein, 2000; Gerstein and Michalski, 1981) describing the use of “Gravitational Clustering” (Wright, 1977): a neuron clustering and visualization tool for identifying groups of neurons with synchronous spiking patterns. This method is based on an analogy of the physics of the gravitational forces governing the dynamics and interactions of macroscopic particles. It treats the N neurons as N particles moving within an N-dimensional space, where charges that influence the attractive and repulsive interactions between particles are dictated by the temporal dynamics of pairwise synchronous spiking activity between neurons. The result is a visualization of particle clusters (and their trajectories) that represent dynamically evolving assemblies of synchronously active neurons. More recent work by Kiani et al. (2015) involves the application of the dimensionality reduction techniques (i.e., multidimensional scaling) to pairwise measures of between-neuron spike rate covariations to detect natural functional modules in a population of pre-arcuate and motor cortex neurons (Kiani et al., 2015). Although the goal of this method is like that of SIMNETS—to the extent that it makes use of single-trial information to group neurons in an unsupervised manner—this approach is applied to binned spike rates and appears to capture the covariation information carried in the absolute firing rates of the neurons’ responses across trials (Kiani et al., 2015), rather than the information carried in the neurons’ intrinsic spike train relational geometry. However, our analysis of the synthetic neurons using temporal codes, as well as rat CA1 neurons demonstrate that SIMNETS can detect a broader range of meaningful information processing motifs that reflect both condition-independent and condition-dependent information carried in the fine temporal structure of spike train outputs.

Yang et al. (2019) identified functional groupings of artificial neurons in a neural network by applying t-SNE to activation-based measures of single neuron selectivity across 20 different tasks (i.e., task variance; Yang et al., 2019). The method is similar to the SIMNETS method in that it projects neurons onto a CS map according to the similarity of measures of output variance. This method requires knowledge of *trial labels* within tasks to group neurons according to the similarities of their changes in selectivity across tasks. By contrast, SIMNETS does not require *a priori* knowledge about the similarities or differences of the information encoded on certain trials (i.e., trial labels), only that the trials are recorded simultaneously. This feature of SIMNETS is particularly valuable when trying to identify groups of functionally similar neurons in experiments with awake and freely moving animals, where the assumption of repeatable perceptual, cognitive, or behavioral states across trials is not always possible.

Several previous studies have used pairwise comparisons between the spike trains of different neurons to identify putative subnetworks or cell assemblies (Aertsen et al., 1989; De Blasi et al., 2019; Gerstein et al., 1985; Grün et al., 2002a; Humphries, 2011; Roudi et al., 2015; Singer and Gray, 1995). As with the gravitational clustering method, these studies have operated under the working hypothesis that the detection of time-series spike time or rate correlations is a signature of a potential functional link (Cole et al., 2016; Roudi et al., 2015; Singer and Gray, 1995; Ts’o et al., 1986). By contrast, SIMNETS identifies neurons with similar informational content even if they exhibit heterogeneous spike train outputs or utilize different encoding timescales (e.g., rate vs. precise spike timing). For the simulated neuron population, SIMNETS successfully identified clusters of the simulated neurons according to their ground-truth functional subnetworks and by increasing the sensitivity of the VP metric, the CS map was able to reveal further sub-groupings within the three identified functional CS clusters that highlighted subtle differences their output space geometries that corresponded to the different encoding timescale. This feature of SIMNETS could be particularly useful for determining if neurons encoded information across heterogenous timescales, e.g., fast vs. slow-timescale inhibitory neurons, or if they compute across a hierarchy of timescales (Cusinato et al., 2022; Fortenberry et al., 2012; Gorchetchnikov and Grossberg, 2007; Honey et al., 2012). Additionally, this feature of SIMNETS enabled us to cluster physiologically and functionally distinct groups of neurons in the CA1 dataset (i.e., place-cells vs. non-place cells), which were not obvious when VP metric was insensitive to spike times (i.e., VP metric q = 0; data not shown).

Capturing the computational similarity between pairs of neurons in a more general way could be accomplished using Information theoretic approaches (Shannon, 1997), which could include estimating the shared or mutual information between the output spike trains of different neurons. Other approaches focus on the asymmetry of the predictive power between variables at different lags, resulting in “directed” estimates of functional connectivity such as Granger Causality, Transfer Entropy, or the Directed Transfer Function (Granger, 1983; Ito et al., 2011; Ursino et al., 2020). These strategies are based on estimating joint probability distributions across the activity patterns of pairs of neurons. A similar approach can be applied to relationships between multiple neurons using Generalized Linear Models (Chen et al., 2009; Truccolo et al., 2005). The number of possible activity patterns is very large, so this type of calculation can be challenging even when relatively large amounts of data are available. Using large amounts of data presents additional problems for this approach because it assumes that relationships between neurons remain relatively constant across the time span used to fit the models. Hence, every additional neuron requires exponentially more data and computing power and analyzing hundreds to thousands of simultaneously recorded neurons becomes intractable.

### 4.3 Mitigating experimenter bias

A considerable amount of research in systems neuroscience has focused on identifying new classes of neurons based on their information-processing properties. The standard approach for many of these experiments involves recording single unit activity while a certain experimental variable of interest is manipulated (for example, providing systematic changes in stimulus features, or eliciting different behavioral responses; Meyer et al., 2016). Standard statistical tests (ANOVA, etc.) are then used to determine if each neuron displays significant changes in firing rate across the experimental conditions. The percentage of significant neurons is usually reported as a functionally distinct “class” of neurons sensitive to the variable of interest. It is common to exclude neurons that do not reach statistical significance or cannot be fit using a predetermined tuning model from further analysis. This approach is prone to both selection and confirmation bias, and ultimately produces “classes” of neurons identified based on arbitrary statistical thresholds imposed on what are likely continuous distributions of properties. The SIMNETS analysis framework is an unsupervised approach to determine if neurons are organized across a functional continuum or are organized into statistically separable functional classes, thereby mitigating the experimenter’s bias inherent in parametric neural discovery methods. The SIMNETS approach is different in that it seeks to organize the neurons into a low dimensional computational similarity (CS) map solely based on the intrinsic structure of their firing rates, without making any *a priori* assumptions about their tuning properties. This makes it possible to detect clusters of neurons with similar informational content and can also reveal gradients of functional properties if they exist. This is not to say that experimental design does not play an important role: the informational content of neuronal spiking is still evaluated within the specific context of the recording. However, SIMNETS does not target specific dependent variables, but instead tries to map the full population of neurons onto a single unified CS map. It is therefore possible to identify ‘orphan’ clusters of neurons that display variations in their spiking related to uncontrolled variables. While assigning a function to an orphan cluster related to an uncontrolled variable may be challenging, it highlights the potential impact of additional variables in the neuronal population, and presents the opportunity to design additional experiments and potentially identify new classes of neurons. In addition to providing a principled way to determine if a consistent organization of information processing modules can be found across sessions and subjects, we believe that the ability to intuitively visualize relationships within networks of neurons will provide a unique perspective leading to new data-driven hypotheses and experimental refinement.

### 4.4 Limitations and considerations

Several important limitations of SIMNETS are worth noting. First, estimates of similarity using spike train metrics require that the time windows of interest be of equal length, making it difficult to compare neural responses with different time courses. This weakness is common to all trial-averaging models commonly used in the literature that we are aware of. It is important to note that the optimal length of the time window will depend on the specific experimental context and should be adjusted on a case-by-case basis, either based on previous work (as done here) or empirically. Using spike train similarity metrics makes it possible to use relatively long time windows without losing information encoded at fine temporal resolution. This feature makes it easier to adopt time windows that fully encompass behavioral events of interest.

Second, although the SIMNETS framework does not require a priori assumptions about the variables potentially encoded by neural activity, experimental design and data selection will still have a direct effect on the results obtained. For example, a set of neurons identified as a functional subnetwork could separate into smaller groups with different computational properties when additional task conditions are added to the analysis. Thus, the functional properties identified using SIMNETS are only valid within the context of the data examined and may not necessarily extrapolate to different experimental conditions. Ultimately, the computational space of the population is only as rich as the experimental design allows (Gao and Ganguli, 2015).

Third, SIMNETS does not provide a means to assess sources of variance. For example, subsets of neurons may exhibit systematic changes in spike train outputs through an experimental session, reward experience, timing, attention, or many other variables which can be revealed by the neural data; however, it will be up to the experimenter to identify their source. Nevertheless, SIMNETS can guide hypothesis generation for experiments that identify these influences.

Fourth, although peak silhouette values are indicative of the optimal number of clusters according to clustering statistics, they should be taken as a guideline and not an absolute measure of the number of subnetworks. The present study focused on a set of diverse recordings from various sources to highlight the performance of the SIMNETS algorithm for different types of data. An in-depth study of potential subnetwork structure using SIMNETS should include many similar datasets, ideally sampling multiple subjects and sessions to properly assess the number of clusters present across many CS maps before drawing conclusions regarding network architecture.

Fifth, it is possible that neuronal subnetworks are constantly re-arranged depending on changing external or internal signals, e.g., ethological demands, task demands, or task stages, or attentional levels. For example, a neuron could potentially exhibit rapid changes in its computational/functional interrelationships with other neurons during processing epochs that are shorter than the selected analysis time window. The current version of the SIMNETS algorithm was not designed to distinguish between rapidly changing sub-network memberships. However, applying the SIMNETS algorithm multiple times over different epochs can help with determining if different network configurations are engaged at different times.

## Data Availability

The datasets presented in this study can be found in online repositories. The SIMNETS software is a C++ optimized MATLAB Package available on GitHub at: https://donoghuelab.github.io/SIMNETS-Analysis-Toolbox/. This includes an interactive MATLAB Live tutorial (.mlx). Additional data was obtained from CRCNS.org data repositories: https://crcns.org/data-sets/vc/pvc-11/about and https://crcns.org/data-sets/hc/hc-5/about-hc-5.
