# Supplementary material for: Mapping the computational similarity of individual neurons within large-scale ensemble recordings using the SIMNETS analysis framework
Source: Front Neurosci. 2025 Aug 14;19:1634652. doi: 10.3389/fnins.2025.1634652 (PMC12391057; doi:10.3389/fnins.2025.1634652)
Supplement: Supplementary file 1 [file Supplementary_file_1.docx]

Supplementary Material for:

Mapping the Computational Similarity of Individual Neurons within Large-scale Ensemble Recordings using the SIMNETS Analysis Framework

# Supplementary Figures


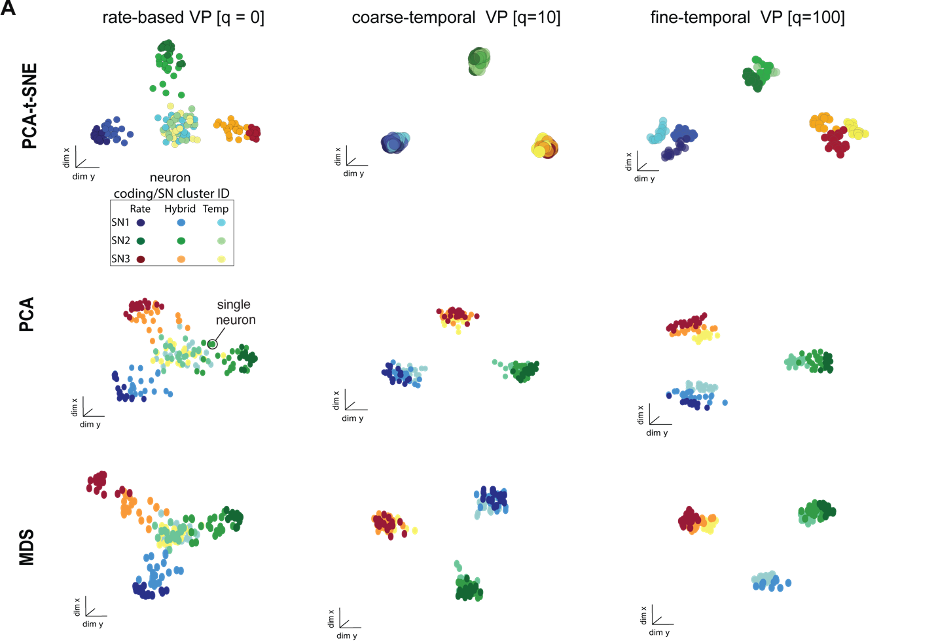


**Supplementary Figure 1.** **Choice of dimensionality reduction method impacts the structure of the low-dimensional CS neuron map.** A) Columns: low-dimensional CS neuron mapping of simulated neuron population dataset for three different VP temporal accuracy values (same as Fig. 5). Rows: the same CS neuron map using PCA-initialized t-SNE (PCA-tSNE) (top row), PCA (middle row), and multidimensional Scaling (MDS, bottom row) (Kruskal and Wish, 1978). Individual points represent individual simulated neurons, and the color labels indicate their simulated ground-truth properties, including coding type and ground-truth CS neuron cluster. PCA is a linear global method; MDS is a local linear method, while PCA-initialized t-SNE combines both linear/global and non-linear/local techniques.

**
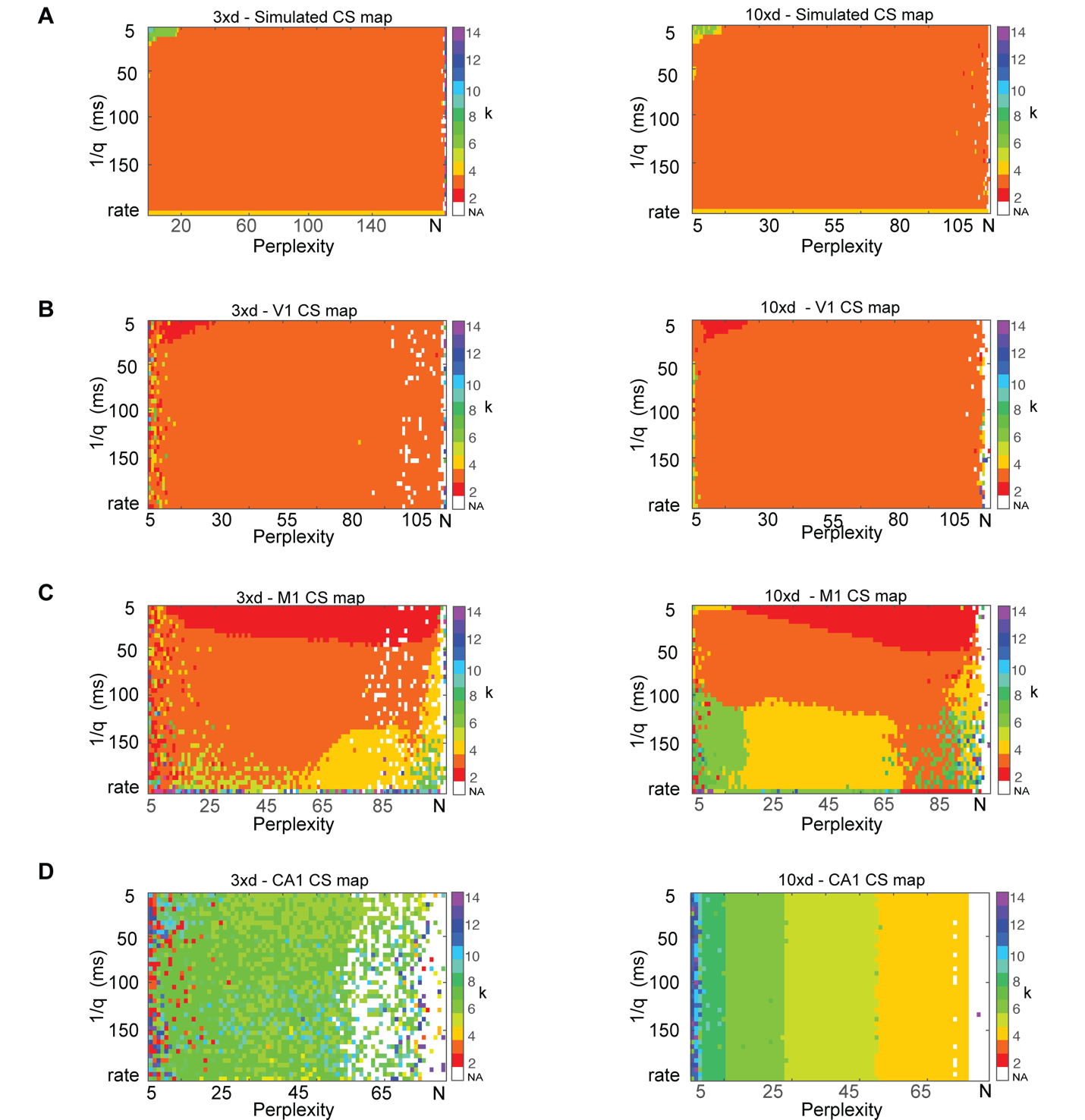
**

**Supplementary Figure 2.** **Stability of cluster evaluation as a function of different SIMNETS parameters and CS map extrinsic dimensionality number.** SIMNETS clusters (*k*) as function of *perplexity* and temporal accuracy (1/*q*) in a 3xN PCA-tSNE projection space (*left*) and a 10-*d* PCA-tSNE projection space (*right*) for the simulated neuron population (N =180, neuron's) (**A**), the V1 neuron population (**B**), the M1 neuron population (**C**), and the Hippocampal CA1 neuron population (**D**). Color bars indicate the optimal number of natural clusters (optimal k-means partitions) within the CS neuron maps, which was determined using a silhouette analysis.


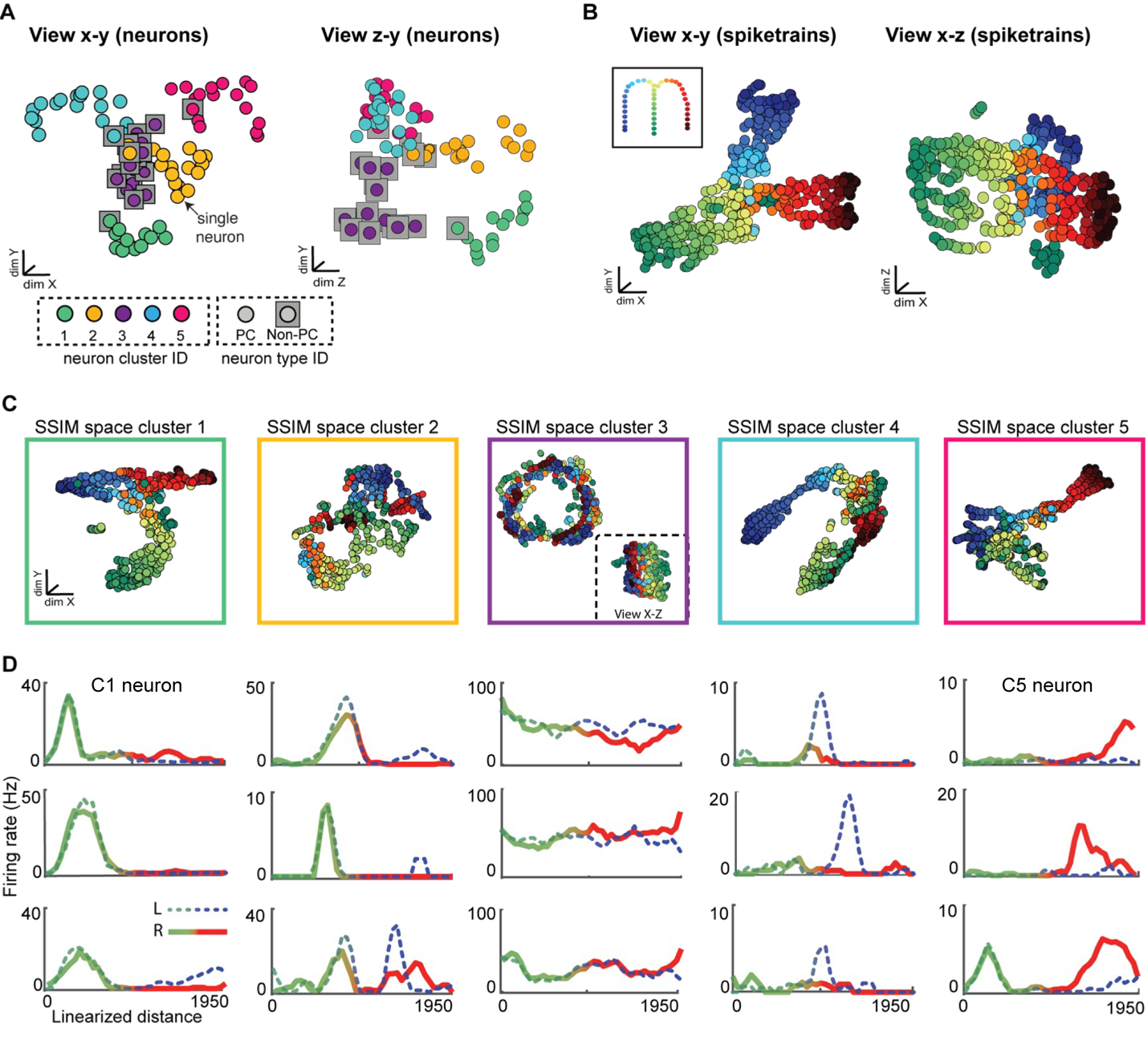


**Supplementary Figure 3.** **Supplementary CA1 Hippocampal analysis provides extended view SIMNETS and SSIMS maps (cont. from Fig. 11).  A)** CA1 population SIMNETS CS map from different viewing angles. *Left*: x-y viewing angle of CS map (same as **Fig. 11A**); *Right***:** z-y viewing angle. Points are neurons; gray squares circumscribe those neurons categorized as non-PC neurons. Color corresponds to the k-means CS neuron clusters. **B)** *Left*: X-Y view of population-level SSIM map. Points are individual spike trains and colors indicate the position in the maze. *Left*: (same as Fig. 11D). *Right***:** x-z viewing angle. **C)** Cluster-level SSIMS maps for all five CS neuron clusters (cluster c3-c5 same as **Fig. 11B**). Outer colored squares correspond to the neuron CS cluster labels in (**A**). The inset cluster c3 neuron (*purple*) provides an additional x-z viewing for the cluster c3 neuron (*purple*) to better highlight its complex shape. **D)** Each column shows three representative single neuron trial-averaged firing-rate functions for the CS neuron clusters c1-c5, respectively. The x-axis corresponds to the linearized distance traveled by the rat along a given arm of the track. The broken green-blue trace corresponds to a left (L) maze run and the solid green-red trace corresponds to right (R) maze run. Rate functions resulted from binning and averaging firing rates across left or right trials and smoothing over with a sliding window.

# Supplementary Methods

**Cluster Validation: Shuffle-based Significance Test** **Silhouette Analysis**

Our CS neuron clusters validation process consists of using a silhouette graphical analysis to determine the natural number of clusters within the empirical CS neuron map (Rousseeuw, 1987) and our novel shuffle-based statistical procedure to verify the statistical validity of these results. Silhouette analysis is used to assess the quality of the clustering obtained from different partitions of a given dataset (in this case, obtained using different values of k for the k-means algorithm). An optimal number of clusters is selected as the partition number, k, that maximizes the average silhouette value, referred to as the Silhouette Coefficient (SC). A silhouette value, h_i_, is a measure of how close a point is to other data points in its assigned cluster as compared to points in other clusters:

|  | $h_{i}=\frac{\left( b_{i}-a_{i} \right)}{max\left( a_{i}{,b}_{i} \right)}$ , | (1) |
| --- | --- | --- |

where a_i_ is the average distance between each point within a given cluster and all other points within that same cluster, and b_i_ is the average distance to each point and all points in other clusters, minimized over all possible cluster configurations. Silhouette values are typically normalized to ([0 to 1]), where a high value indicates that a given point is well matched to its own cluster and far from neighboring clusters. In general, a maximized average silhouette below 0.25 indicates data that are not structured while a value below 0.5 would indicate poor or potentially spurious clusters. In the next section, we outline a procedure for testing the statistical significance of the optimal cluster number identified using the Silhouette analysis.

We developed a significance test for the purpose of determining the likelihood of detecting a given number of clusters by chance using silhouette analysis under the null hypothesis that there is no genuine covariation relationship between the inherent structures of the SSIM matrices. The significance test involves generating a null distribution of silhouette values based on shuffled data across a range of partition (k) values. In SIMNETS, computational similarities are captured by the pairwise measures of correlation between each neuron’s respective spike train distances values in the single neuron SSIM matrices. Our test relies on a shuffling procedure that destroys the pairwise dependencies between the SSIM matrices, and subsequently, any significant measures of correlation in the CS neuron matrix. This approach is inspired by the Mantel test, a permutation-based procedure that tests the significance of the observed correlation between two symmetric matrices (Legendre, 2000; Mantel, 1968). The intuition of a Mantel test is that if a significant relationship exists between the values of matrix A and matrix B, then randomizing the rows and columns of one matrix will destroy any existing dependencies. As a result, the correlation between the shuffled matrix pair will tend to be lower than the original correlation value observed between the un-shuffled matrix pair. The probability of observing r(A, B) is then calculated as the proportion of permutations for which the shuffled correlation measures are smaller than or equal to r(A, B). Here, we carry out a similar permutation operation on the SSIM matrices, in that we destroy any dependencies that exist between the matrices; however, we use the average silhouette value as the test statistic, rather than the correlation values, as is the case with the Mantel test.

The procedure involves symmetrically shuffling the rows/columns of each N SSIM matrix separately and re-calculating the pairwise correlations between the SSIM matrices to generate a new NxN CS matrix. This NxN CS matrix is then transformed into a new CS map using t-SNE, and a new set of silhouette values is calculated for the range of tested partition values. This procedure is repeated to generate a null distribution of average silhouette values that are approximately normally distributed (e.g., 1000+ iterations). If the observed maximized average silhouette value, SC, falls above the empirically calculated (1-α)100% confidence interval, then the detected number of clusters is considered statistically meaningful.

**Table 1: Summary of SIMNETS algorithm Inputs/Outputs across datasets.**

| **Symbol** | **Simulated Dataset** | **V1** **Dataset** | **M1**  **Dataset** | **CA1**  **Dataset** |
| --- | --- | --- | --- | --- |
| ***N*** | 180 | 112 | 103 | 80 |
| ***T*** | 30 | 360 | 114 | 17 |
| ***S*** | 30 | 360 | 114 | 442 |
| ***q*** | [0, 10, 200] | 20 | 10 | 35 |
| ***perp*** | 50 | 30 | 20 | 15 |
| ***d*** | 3 | 3 | 3 | 3 |
| **SC** | 0.5, 0.99, 0.95 | 0.89 | 0.68 | 0.70 |
| **K_(SC)_** | 4, 3, 3 | 3 | 3 | 5 |

*Abbreviations: N, neuron number; T, trial number; S, number of spike trains; q, temporal accuracy parameter; perplexity, t-SNE “number of nearest neighbors” parameter; d, dimensions of CS map display plot; map; K_(SC)_, optimal number of statistically significant clusters; SC, silhouette coefficient.*

**Simulated Dataset – Data Simulation and Analysis**

Spike train Simulation We simulated the spiking activity of a population of N = 180 synthetic neurons that consisted of 3 computationally distinct subnetworks (SN1, SN2, SN3) of 60 neurons. Each subnetwork was designed to produce similar spike trains for two non-modulating conditions, referred to as the “baseline” conditions, and a different pattern for a third condition, referred to as the “modulating” condition. For example, subnetwork SN1 was modulated during condition A and exhibited the same baseline activity spike pattern during both conditions B and C, whereas subnetwork SN1 was modulated during condition B and exhibited the same baseline spike pattern during conditions A and C, etc. The neurons within each subnetwork could be further divided into three sub-groups of n = 20 neurons, where each sub-group altered their spike-train patterns between the active and baseline states according to one of three different encoding strategies:

i. Rate coding: firing rate increased by 50% for the modulating condition (all spike times were randomly chosen)

ii. Temporal coding: the two baseline conditions and the modulating condition were associated with specific (randomly generated) temporal sequences of spikes. The number of spikes was kept constant across baseline and modulating conditions. Spike times were jittered by +/- 5 ms for each trial.

iii. Mixed temporal/rate coding: similar to the temporal coding, but additionally, the modulating condition included 25% more spikes.

To simulate stochastic variation in spiking patterns, 50% of the spikes were randomly removed for each condition. A total of 30 seconds of simulated recording time was generated, with the trial condition changing every second between A, B, and C patterns. This example dataset is included in the SIMNETS software package included with this submission. For MATLAB Tutorial, see:

[https://donoghuelab.github.io/SIMNETS-Analysis-Toolbox/SIMNETS.mlx](https://donoghuelab.github.io/SIMNETS-Analysis-Toolbox/)

**Neural Datasets – Task Description and Data Analysis**

***Primate Primary Visual Cortex Dataset***

**Task Description.** We analyzed a previously described dataset of 112 primary visual (V1) single-units (which we refer to as neurons) recorded in an anesthetized Macaca Fascicularis using a 96-channel microelectrode array (Kohn and Smith, 2016; Smith and Kohn, 2008). We analyzed the data from a single subject (monkey 3, single session). Briefly, sinusoidal gratings were presented at 6 different orientations 𝜃 = {0°, 30°, 60°, 90°, 120°, 150°} and 2 drift directions (rightward and leftward drift, orthogonal to orientation). Each stimulus was presented 112 times for 1.28 seconds. The position and size of the stimuli was sufficient to cover the receptive fields of all recorded neurons. For more details on the task design and data pre-processing, *see* Kohn and Smith, 2016 and Smith and Kohn, 2008, or go to the CRCNS.org data repository: <https://crcns.org/data-sets/vc/pvc-11/about>).

**Single Neuron Tuning Analysis.** The single neuron analyses described were applied to the same set of spike trains used in the SIMNETS analysis. This involved extracting 1 second of spiking data from the first 30 repetitions of each stimulus (S = 360, spike trains), starting 0.28 seconds after stimulus onset. Only a small fraction of the total number of recorded trials was used in the analysis (25%) as we wanted to demonstrate SIMNETS ability to cluster neurons in datasets where only a small number of trials are available. These analyses were carried out independently from the SIMNETS analysis and were not used to guide the SIMNETS algorithm in any way.

We characterized the preferred orientation of each V1 neuron by fitting a Gaussian distribution to the firing rate function R:

|  | $R\left( \theta\right)=A^.{exp}^{\frac{-\left( \theta-\mu\right)^{2}}{2\sigma^{2}}}$ , | (2) |
| --- | --- | --- |

where is the stimulus orientation, Â is the peak response, μ the mean, and σ^2^ is the variance of the Gaussian. The function takes on a maximum value at *θ* = μ, for θ = [0, 180), which corresponds to the neuron’s preferred orientation. Drift-direction was not explicitly repotted in this work, but it was taken into consideration by calculating preferred orientation separately for the different drift directions (i.e., *θ* = [0, 180) and *θ* = [180, 360)). Summary statistics of drift direction were not reported but an example neuron with direction-of-motion dependent orientation selectivity is presented in the main text (Fig. 7C, see example n36 in CS cluster 3). We calculated the normalized “peak-to-trough” of the orientation response function, the orientation index (OI), as an indicator of orientation tuning strength:

*OI* = (*R_pref_ori_* − *R_orth_*)/*R_pref_ori_*, (3)

where *R_pref_ori_* is the “peak response” of the orientation tuning function (See: eqt. 2, equivalent to R(θ), and *R_orth_* is the “trough response” at the orthogonal angle (or function minimum) of the tuning function (Mazurek et al., 2014).

***V1 SIMNETS CS Map Characterization*** We used a circular-linear correlation (r_cl_) analysis to assess SIMNETS’ ability to organize neurons according to their computational properties (Berens, 2009). The correlation between each neuron’s preferred orientation and its location along each dimension in the low dimensional map y_i_ was calculated using:

$r_{\theta,y}=\frac{cov\left( \theta_{i},y_{i} \right)}{\sigma_{A}\sigma_{B}},$ (4)

, where 𝜎𝐴 and 𝜎𝐵 are the standard deviations of the neurons’ preferred orientations and y represents the neurons’ locations in the map. A high correlation value indicates a strong relationship between a neuron’s preferred orientation/direction and map location and demonstrates that functionally similar neurons were mapped to nearby regions of the map. The r_cl_ value for the dimension with the highest value was reported.

***Primate Primary Motor Cortex Dataset***

**Task Description.** SIMNETS was applied to the previously described dataset of *Macaca mulatta* primary motor (M1) cortex neurons (i.e., single-units) recorded during a planar 8-direction reaching task (Rao and Donoghue, 2014). The single-unit activity was simultaneously recorded from the upper limb area of the primary motor cortex using a chronically implanted microelectrode array. The monkey was operantly trained to move a cursor that matched its hand location to targets projected onto a horizontal reflective surface. A visual cue was used to signal movement direction during a variable duration instructed delay period (1 – 1.6 s) to one of eight radially distributed targets on the screen with the associated reach angles of 𝜑 = {0°, 45°, 90°, 135°, 180°, 225°, 270°, 315°}. At the end of the instructed delay period, the central target was extinguished, instructing the monkey to reach towards the previously cued target.

**Single Neuron Tuning Analysis.** We analyzed 1 second of neural data from correct trials (S = 114), starting 0.1 second before movement onset. Characterization of the detected SIMNETS clusters is like that described in the previous section. We characterized the preferred movement direction of each M1 neuron by fitting a von Mises distribution to the firing rate function R:

𝑟(𝜑) = 𝛽 + ℎ. 𝑒𝑥𝑝(ℎ. 𝑐𝑜𝑠(𝜑 − 𝜇)), (5)

where 𝛽 is the offset of the function, h is the depth of the tuning, is the reach angle and μ is preferred reach direction of the cell (Mardia and Zemroch, 1975). The function takes on a maximum value at μ, which corresponds to the neuron’s preferred direction. The tuning functions were then used to calculate each neuron’s tuning depth, or the reach direction index (RDI). This was calculated as the normalized peak-to-trough responses using an equation of similar form to the V1 neuron OI (See Equation (3)).

***Rat Hippocampal CA1*** ***Dataset***

**Task Description.** We applied SIMNETS to a previously described dataset of rat hippocampal neurons made publicly available by the Collaborative Research in Computational Neuroscience (CRCNS.ORG) data-sharing repository (Pastalkova et al., 2015, 2008). We analyzed the data from a single subject rat during one session (dataset: hc-5 01_maze06_MS.002). The neurons were simultaneously recorded from the CA1 hippocampal region using multi-site silicon probes while the rat performed a spatial navigation task in a maze. Briefly, the rat was trained to run through the arms of a “figure-8” maze in a left/right alternating manner to receive a reward. The left/right track runs were interleaved with a wheel-run period that functionally served as a memory delay-period. The rat performed T = 17 correct trials (Right= 8, left trials; T*_left_*= 9, right trials), taking on average 4.3 seconds to reach the rewards located at either end of the arms. The rat’s path along each arm of the track was linearized and divided into small spatial bins (80cm) for the SIMNETS analysis, respectively. For additional details go to CRCNS.org repository: https://crcns.org/data-sets/hc/hc-5/about-hc-5).

**Single Neuron Place Field Analysis.** The rat’s path along each arm of the track was linearized and divided into 80 mm spatial bins when generating the spatial firing field maps. Bins corresponding to reward locations and the inter-trial activity were excluded from the analysis, leaving a total of 390 bins for each of the left and right trajectories, where the first 19 spatial bins were common to both trajectories. We generated a separate spatial firing map for the left and right trajectories of each neuron by dividing the number of spikes in the i-th bin by the rat’s occupancy time t_i_ and used a Gaussian kernel (width = 3 bins/150 mm) to smooth across the firing rates in each bin. Neurons that did not exhibit a 5 Hz firing rate in at least 1 spatial bin were not included in the analysis, leaving a total of N = 80 neurons. We characterized the neurons as non-place cells (n = 20, non-PC) or place cells (n = 60, PC) based on their spatial firing properties and an information-theoretic measure of the spatial information in their spikes (Eqt. 6) (Skaggs et al., 1992a). Neurons were classified as having place cell-like activity if the firing rate in three contiguous bins exceeded the mean of all other firing fields by 20% (Jeffery et al., 1997; Skaggs et al., 1992)(using 2.5 STD of the out-of-field firing rate produced similar results) and if their information content exceeded 0.5 bits/spike on either the left or right trajectories (Skaggs et al., 1992). The spatial information metric, I_spike_, is a measure of the extent to which a neuron’s spiking activity can be used to predict the rat’s position along the track. The spatial information content of the neuron (measured in bits/spike) is defined as:

$I_{spike}=\sum_{i=1}^{l} P_{i}\frac{v_{i}}{V}{log}_{2}\frac{v_{i}}{V},$ (6)

where Pi is the occupancy probability, 𝑣𝑖 is the firing rate in the i-th bin, and V is the overall mean firing rate of the cell across all bins in trajectory.
